# The Kinase Complex mTOR Complex 2 Promotes the Follicular Migration and Functional Maturation of Differentiated Follicular Helper CD4^+^ T Cells During Viral Infection

**DOI:** 10.3389/fimmu.2018.01127

**Published:** 2018-05-23

**Authors:** Yaxing Hao, Yifei Wang, Xiaobing Liu, Xia Yang, Pengcheng Wang, Qin Tian, Qiang Bai, Xiangyu Chen, Zhirong Li, Jialin Wu, Zhunyi Xie, Xinyuan Zhou, Yuyang Zhou, Zhinan Yin, Yuzhang Wu, Lilin Ye

**Affiliations:** ^1^Institute of Immunology, Third Military Medical University, Chongqing, China; ^2^The First Affiliated Hospital, Biomedical Translational Research Institute, Guangdong Province Key Laboratory of Molecular Immunology and Antibody Engineering, Jinan University, Guangzhou, China; ^3^State Key Laboratory of Biotherapy, Collaborative Innovation Center for Biotherapy, West China Hospital, Sichuan University, Chengdu, China

**Keywords:** mechanistic target of rapamycin complex 2, follicular helper T cells, germinal center, B cells, acute viral infection

## Abstract

Follicular helper CD4^+^ T (T_FH_) cells are critical for optimal B-cell-mediated humoral immunity by initiating, fueling, and sustaining germinal center reactions. The differentiation of T_FH_ cells relies on multiple intrinsic and extrinsic factors; however, the details by which these factors are integrated to coordinate T_FH_ differentiation are largely unknown. In this study, using a mouse model of acute lymphocytic choriomeningitis virus (LCMV) viral infection, we demonstrate that mTOR complex 2 (mTORC2) kinase integrates TCR signaling and ICOS-mediated co-stimulation to promote late differentiation and functional maturation of virus-specific T_FH_ cells. Specifically, mTORC2 functions to maintain T_FH_ lineage specifications, including phenotypes, migratory characteristics, and functional properties. Thus, our results highlight the importance of mTORC2 in guarding T_FH_ phenotypic and functional maturation.

## Introduction

Effective humoral immunity protects individuals from invading pathogens by producing high-affinity, class-switched antibodies, which require cooperation between pathogen-specific B cells and follicular helper CD4^+^ T (T_FH_) cells. T_FH_ cells were originally defined as a unique helper CD4^+^ subset characterized by high expression of the chemokine receptor CXCR5, which facilitates T_FH_ cell migration toward B cell follicles (1–6), where they interact with and assist cognate B cells. T_FH_ cells provide essential signals to B cells, including engagement of the inducible T cell co-stimulator ICOS, the ligand for the costimulatory receptor CD40, T cell inhibitory receptor PD-1, interleukin 21 (IL-21), and interleukin 4 (IL-4) (7), leading to the formation and maintenance of germinal centers (GCs), in which B cells undergo somatic hypermutation, antibody affinity maturation, and final differentiation into long-lived memory B cells and plasma cells (8).

Follicular helper CD4^+^ T cell differentiation is a multistage process that is tightly controlled by multiple factors (7). In acute viral infection, dendritic cells (DCs) initiate T_FH_ programming by priming antigen-specific CD4^+^ T cells for activation (9). The activated CD4^+^ T cells, with upregulated CXCR5 and Bcl-6 but downregulated Blimp-1 expression, differentiate toward T_FH_ cells (10–12). The T_FH_ precursors are generated as early as 2 days after activation (13), which has recently been proven to be mediated by the transcription factor T cell factor-1 (TCF-1) and achaete scute homolog-2 (ASCL2) (14–17). TCF-1 promotes early T_FH_ cell differentiation by upregulating Bcl-6 but repressing Blimp1 expression (14), while ASCL2 participates in early T_FH_ induction by increasing the follicular homing ability of T_FH_ cells (17). These early differentiated T_FH_ cells subsequently migrate to the T cell–B cell border and acquire full polarization into GC T_FH_ cells with maximal functions upon engagement of B cells (18–21). Of note, initiation of T_FH_ differentiation requires priming from DCs but not B cells (7, 9, 22, 23), while full commitment and maintenance of T_FH_ cells depend on the presence of cognate B cells (7, 18, 23). Unlike early T_FH_ early polarization, underlying mechanisms during complete differentiation and functional maturation of T_FH_ cells in the B cell-dependent phase are poorly understood.

The mechanistic target of rapamycin (mTOR) is an evolutionarily conserved serine/threonine kinase that is involved in diverse cellular processes, including cell growth, proliferation, differentiation, metabolism, and survival, by sensing and integrating environmental cues (24). The mTOR kinase exists in two distinct complexes named mTOR complex 1 (mTORC1) and mTOR complex 2 (mTORC2), which are defined by scaffolding subunit regulatory-associated protein of mTOR (*Raptor*) and rapamycin-insensitive companion of mTOR (*Rictor*), respectively (25, 26). mTORC1 has been reported to regulate protein translation and glucose and lipid metabolism by phosphorylating the downstream targets ribosomal protein S6 kinase and eIF4E-binding protein 1 (4E-BP1) (27–29). mTORC2 mainly engages in the regulation of cell survival, metabolism, and cytoskeletal organization by phosphorylating many AGC kinases, including AKT (at position Ser473), SGK1, and PKC-α (30). Both mTORC1 and mTORC2 have been reported to participate in a variety of T cell immune responses (31). mTORC1 signaling promotes the differentiation of T_H_1 and T_H_17, whereas it inhibits the differentiation and suppressor functions of T_Reg_ cells (32–37). Additionally, the interleukin-2 (IL-2)-mTORC1 signaling axis promotes T_H_1 but inhibits T_FH_ cell differentiation to orchestrate the reciprocal balance between T_H_1 and T_FH_ cell fates during acute viral infection (38). Additionally, mTORC1 has been reported to regulate follicular regulatory T (T_FR_) cell differentiation from conventional regulatory T cells (39). In contrast to mTORC1, the knowledge about mTORC2 in the regulation of T cell immunity is quite limited. It is known that mTORC2 signaling favors T_H_2 differentiation (32, 40). Recently, two groups have reported that mTORC2 is essential for T_FH_ cell differentiation at the steady state in Peyer’s patches (PPs) and upon protein immunization or viral infection (41, 42). However, it remains unknown whether mTORC2 selectively regulates early fate commitment or later lineage maintenance of T_FH_ cells, or both. Additionally, whether mTORC2 regulates the effector functions of differentiated T_FH_ cells remains to be investigated.

Here, we investigate the role of mTORC2 in regulating T_FH_ differentiation at early and late stages, as well as the effector function of T_FH_ cells in response to acute viral infection. We use lymphocytic choriomeningitis virus (LCMV) to establish a mouse model of acute viral infection, in which virus - specific CD4^+^ T cells primarily differentiate into T_FH_ and T_H_1 effector cells (43). Our findings demonstrate that mTORC2 signaling is selectively critical for T_FH_ differentiation in the late stage (4–8 days), but not early fate commitment (1–3 days). Moreover, mTORC2 plays an essential role in mediating the effector function of T_FH_ cells to assist B cells, which is accomplished by regulating the T_FH_ transcriptional program and migratory ability toward B cell follicles.

## Materials and Methods

### Mice, Virus, and Immunization

*Rictor*^fl/fl^, *Cd4*-Cre transgenic, and C57BL/6J (CD45.2^+^ and CD45.1^+^) mice were obtained from the Jackson Laboratory. *Sh2d1a^−/−^* (*Sap^−/−^*) mice were provided by Dr. Hai Qi (Tsinghua University). SMARTA (CD45.1^+^) transgenic mice and the LCMV Armstrong strain were provided by Dr. Rafi Ahmed (Emory University). The mice were infected with 2 × 10^5^ plaque-forming units (PFU) of LCMV Armstrong at 6–10 weeks of age, and both sexes were included without randomization or “blinding.” Bone marrow (BM) chimeras were infected after 8–10 weeks of reconstitution. To establish bacterial infection, mice were intravenously infected with 1 × 10^6^ colony-forming units of *Listeria monocytogenes* expressing LCMV glycoprotein-specific I-Ab-restricted CD4^+^ T cell epitope gp61-80 (LM-gp61), that was created from vector strain1 (44). 4-Hydroxy-3-nitrophenylacetyl-conjugated ovalbumin (NP-OVA) (N-5051-100, Biosearch Technology) was 1:1 emulsified with Complete Freund’s Adjuvants (F5881, Sigma) and immunized mice subcutaneously of 100 µg per mouse. All immunized mice were housed in accordance with institutional biosafety regulations of the Third Military Medical University. All mouse experiments were performed in accordance with the guidelines of the Institutional Animal Care and Use Committees of the Third Military Medical University.

### Flow Cytometry and Antibodies

Major histocompatibility complex class II (I-A^b^) tetramer specific for the LCMV epitope of glycoprotein amino acids 66–77 was provided by the tetramer core facility of the US National Institutes of Health (Emory). The antibodies used for flow cytometry are listed in Table S1 in Supplementary Material. Surface staining was performed in PBS containing 2% FBS. CXCR5 staining was performed using purified anti-CXCR5 (BD Biosciences) for 1 h at 4°C, followed by biotinylated anti-rat immunoglobulin G (IgG) (Jackson Immunoresearch) and then fluorescently labeled streptavidin (eBioscience) for 30 min on ice. Staining was performed in PBS containing 0.5% BSA, 2% FCS, and 2% normal mouse serum. Staining for Bcl-6, c-Maf, TCF-1, IgG1, IgG2a, and Foxp3 was performed with the Foxp3/Transcription Factor Staining Buffer Set (00-5523, eBioscience). Major histocompatibility complex class II tetramer staining was performed by incubation of the tetramer with cells for 1 h at 37°C. For detection of phosphorylated mTOR signaling proteins, lymphocytes were first stained with surface markers and then were stimulated with anti-CD3 (2 µg/ml, 100302, Biolegend), anti-CD28 (0.5 µg/ml, 102102, Biolegend), anti-ICOS (2 µg/ml, 14-9949-82, eBioscience), gp61–80 peptide (2 µg/ml), or CXCL13 (4 µg/ml, 4583906, Biolegend) at 37°C for 1 h. Stimulated cells were immediately fixed with Phosflow Lyse/Fix buffer (558049, BD Biosciences), followed by permeabilization with Phosflow Perm buffer I (557885, Biosciences) and staining with primary unconjugated antibodies against p-S6 (Ser 235/236) (D57.2.2E, Cell Signaling Technology) and p-AKT (Ser 473) (#4060S, Cell Signaling Technology). Next, primary unconjugated antibodies were detected by secondary staining with anti-rabbit IgG A488 antibody (A21206, Invitrogen) or anti-rabbit IgG A647 antibody (#4414S, Cell Signaling Technology). Flow cytometry data were acquired with a FACS Canto II (BD Biosciences) and were analyzed with FlowJo software (Tree star, Ashland, OR, USA).

### Retroviral Constructs and Transduction

The humanized-*Cre* (hCre) coding sequences were amplified and cloned into the vectors MIGR1 (MSCV-IRES-GFP). Retroviruses were packaged by transfection of plat-E cells with the retroviral vectors along with plasmid pCL^eco^. SMARTA cells were activated *in vivo* by injection of 200 µg of peptide (LCMV glycoprotein amino acids 61–80) into SMARTA mice. After 18 h, activated SMARTA cells were purified by negative selection with BeaverBeads Mag500 Streptavidin Matrix (22302, Beaver) and then “spin-infected” for 90 min at 37°C by centrifugation (800 × *g*) with freshly harvested retrovirus supernatants, 8 µg/ml polybrene (H9268, Sigma-Aldrich), and 20 ng/ml of IL-2 (130-098-221, Miltenyi Biotec). Then, the transduced SMARTA cells were transferred into recipient mice, followed by infection of the hosts with LCMV.

### Adoptive Transfer

A total of 1 × 10^6^ (for analysis at day 3) or 2 × 10^4^ (for analysis at day 8) retrovirus-transduced SMARTA (CD45.1^+^) cells were adoptively transferred into naive C57BL/6J (CD45.2^+^) mice, which were infected intravenously with 2 × 10^6^ PFU (day 3) or infected intraperitoneally with 2 × 10^5^ PFU (day 8) of the LCMV Armstrong strain on the following day. For assessment of mTOR activity kinetics, a total of 2 × 10^6^ (for analysis at day 2) or 4 × 10^4^ (for analysis at day 5) or 2 × 10^4^ (for analysis at day 8) naive SMARTA (CD45.1^+^) cells were adoptively transferred into naive C57BL/6J (CD45.2^+^) mice, which were infected intravenously with 2 × 10^6^ PFU (for analysis at day 2) or infected intraperitoneally with 2 × 10^5^ PFU (for analysis at day 5 and 8) of the LCMV Armstrong strain on the following day. For evaluation of T_FH_ cell function, 3 × 10^6^ sorted T_FH_ cells from *Rictor^−/−^* or WT mice (CD45.1^+^) were adoptively transferred into *Sap^−/−^* recipient mice (CD45.2^+^) which were infected with LCMV 1 day before cell transfer and then the hosts were analyzed on day 6 after cell transfer.

### Enzyme-Linked Immunosorbent and Enzyme-Linked Immunospot Assay

Lymphocytic choriomeningitis virus-specific IgG and antibody-secreting cells (ASCs) were measured by enzyme-linked immunosorbent assay (ELISA) and enzyme-linked immunospot (ELISPOT) assay, respectively, which has been described (45, 46).

### Generation of Bone Marrow Chimeras

For each chimera, 5 × 10^6^ BM cells of a 4:6 mixture derived from *Rictor^−/−^* or *Rictor*^fl/fl^ (CD45.2^+^) mice and C57BL/6J (CD45.1^+^) mice were intravenously transferred into lethally irradiated (2 doses of 550 rads each) C57BL/6J (CD45.1^+^) recipients. Recipient mice were allowed 8–10 weeks for reconstitution before infection with LCMV.

### Immunofluorescence Staining

Tissues immersed in OCT were quickly frozen in liquid nitrogen and cut into 7-µm-thick sections. Frozen tissue sections were fixed in cold acetone for 10 min at −20°C, blocked with 5% BSA and 1:100 Fc-blocker in PBS, and stained with biotin-IgD, FITC-labeled anti-GL7, and PE-labeled anti-CD4, followed by Alex 650 dye-labeled avidin. After each step, the slides were washed at least three times with PBS. Coverslips were mounted on slides using an antifade kit (BOSTER) and then examined using a Zeiss LSM 800 confocal fluorescence microscope. The images were processed with LSM Image Examiner software (Zeiss).

### Microarray and Bioinformatics Analysis

Isolation of T_FH_ cells from WT and *Rictor^−/−^* mice at day 8 after infection has been described previously (14). Total RNA was extracted according to the TRIzol reagent protocol (Life Technologies) and submitted to CapitalBio for microarray analysis. Gene-set-enrichment analysis (GSEA) software (Broad Institute) was used for analysis (47). The data discussed in this publication have been deposited in NCBI’s Gene Expression Omnibus (48) and are accessible through GEO Series accession number GSE111536 (https://www.ncbi.nlm.nih.gov/geo/query/acc.cgi?acc=GSE111536).

### Quantitative RT-PCR

For comparison of gene expression in T_FH_ cells from *Rictor^−/−^* and WT mice, the cells were sorted and subsequently lysed in TRIzol LS reagent (10296; Life Technologies). Total RNA was extracted and reverse-transcribed with a RevertAid H Minus First-Strand cDNA Synthesis Kit (K1632; Thermo Scientific). The resulting cDNA was analyzed for expression of various genes with the SYBR Green PCR kit (208054, QIAGEN) on a CFX96 Touch Real-Time System (Bio-Rad) and the appropriate primers for “test genes” (Table S2 in Supplementary Material).

### Transwell Migration Chemotaxis Assay

For enrichment of CD4^+^ T cells, total splenocyte samples from WT and *Rictor^−/−^* mice at day 8 after infection with LCMV were subjected to depletion of cells that were positive for lineage markers (Lin^+^ cells) using biotin-conjugated antibodies [anti-CD8 (53–6.7), anti-B220 (RA3-6B2), anti-CD11c (N418), anti-Gr-1 (RB6-8C5), anti-TER119 (TER-119), and anti-NK1.1 (PK136), all from Biolegend] coupled to the BeaverBeads Mag500 Streptavidin Matrix (22302, Beaver). The surfaces of the Lin^−^ cells were then stained with anti-CD4, anti-CD44, anti-GITR, anti-CD25, and anti-CXCR5 to identify T_FH_ cells. Next, 4 × 10^5^ T_FH_ cells from WT or *Rictor^−/−^* mice were loaded into the upper chamber of a 24-well transwell plate (5-µm pore, Corning), and 600 µl of chemotaxis medium supplemented with or without the CXCL13 (4 µg/ml, 4583906, Biolegend) was added to the lower chamber. The cells were allowed to migrate for 3 h in a 5% CO_2_ incubator at 37°C. Then, all the migrated cells were collected from the lower chamber, and the numbers of migrated T_FH_ cells were determined by flow cytometry (FACS Canto II). Based on the absolute number of T_FH_ cells, the “net migration (% of input)” was calculated as follows: Net migration (% of input) = (# of migrated T_FH_ cells to CXCL13 − # of migrated T_FH_ cells in the absence of CXCL13)/(# of T_FH_ cells in the input sample).

### Conjugate Adhesion Assay

B cells were activated *in vivo* by injection of 30 µg of LPS (ALX-581-008-Loo2, Enzo Life Sciences) into naive C57BL/6J mice and purified by negative selection with magnetic beads (22302, Beaver) after 18 h. *Rictor*^fl/fl^-*Cd4*-Cre-SMARTA (*Rictor^−/–^* SMARTA) cells and WT SMARTA (CD45.1^+^) cells were adoptively transferred into naive C57BL/6J (CD45.2^+^) mice, following intraperitoneal infection with 2 × 10^5^ PFU of the LCMV Armstrong strain. *Rictor^−/−^* and WT SMARTA T_FH_ cells were sorted from *Rictor^−/−^* SMARTA and WT SMARTA chimeras, respectively, at day 8 after LCMV infection. Then, 2 × 10^5^ *Rictor^−/−^* and WT SMARTA T_FH_ cells were incubated for 1 h at 37°C in 96-well U-bottom plate with 8 × 10^5^ LPS-activated B cells pulsed with gp61–80 peptide (LCMV glycoprotein amino acids 61–80). The frequency of T cell–B cell conjugates was quantified by flow cytometry as CD4^+^B220^+^. I-A^b^ gp66-77 tetramer^+^ T_FH_ cells were also used to perform the adhesion assay, and 5 × 10^4^ I-A^b^ gp66–77 tetramer^+^ T_FH_ cells were incubated for 1 h at 37°C in 96-well U-bottom plate with 2 × 10^5^ LPS-activated B cells pulsed with gp61-80 peptide. The frequency of T cell–B cell conjugates was quantified by flow cytometry as I-A^b^ gp66-77 tetramer^+^ CD4^+^ B220^+^.

### *In Vitro* T_FH_ Function Assay

Follicular helper CD4^+^ T (T_FH_) cells were sorted from WT or *Rictor^−/−^* mice, and B cells were sorted from C57BL/6J mice at day 8 after LCMV infection. U-bottom 96-well plates were seeded with 5 × 10^4^ B cells alone, 5 × 10^4^ B cells and 3 × 10^4^ wild-type T_FH_ cells or 5 × 10^4^ B cells and 3 × 10^4^ *Rictor^−/−^* T_FH_ cells, and then supplemented with 200 µl of RPMI medium (10% FBS, 1 × Pen–Strep, l-glutamine) containing anti-CD3 (2 µg/ml, 100302, Biolegend) and anti-IgM (5 µg/ml, 115-006-075, Jackson Immunoresearch). The plates were cultured for 4 days at 37°C, followed by FACS staining using anti-GL7, anti-IgG1, anti-IgG2a, anti-I-A/I-E, anti-CD19, and anti-CD4.

### Statistical Analysis

Statistical analysis was conducted with Prism 6.0 software (GraphPad). An unpaired two-tailed *t*-test with 95% confidence interval was used for calculation of *P* values. For retroviral transduction and BM chimera experiments, a paired two-tailed *t*-test with 95% confidence interval was used for calculation of *P* values. For microarray analysis, we used an unpaired one-tailed *t*-test with 95% confidence interval for calculation of *P* values. For *in vivo* and *in vitro* T_FH_ function assay and T cell–B cell adhesion assay, we used the one-way ANOVA with multiple comparisons for calculation of *P* values.

## Results

### mTORC2 Signaling Is Elevated in T_FH_ Cells and Activated by ICOS and CD3

**Figure 1:**
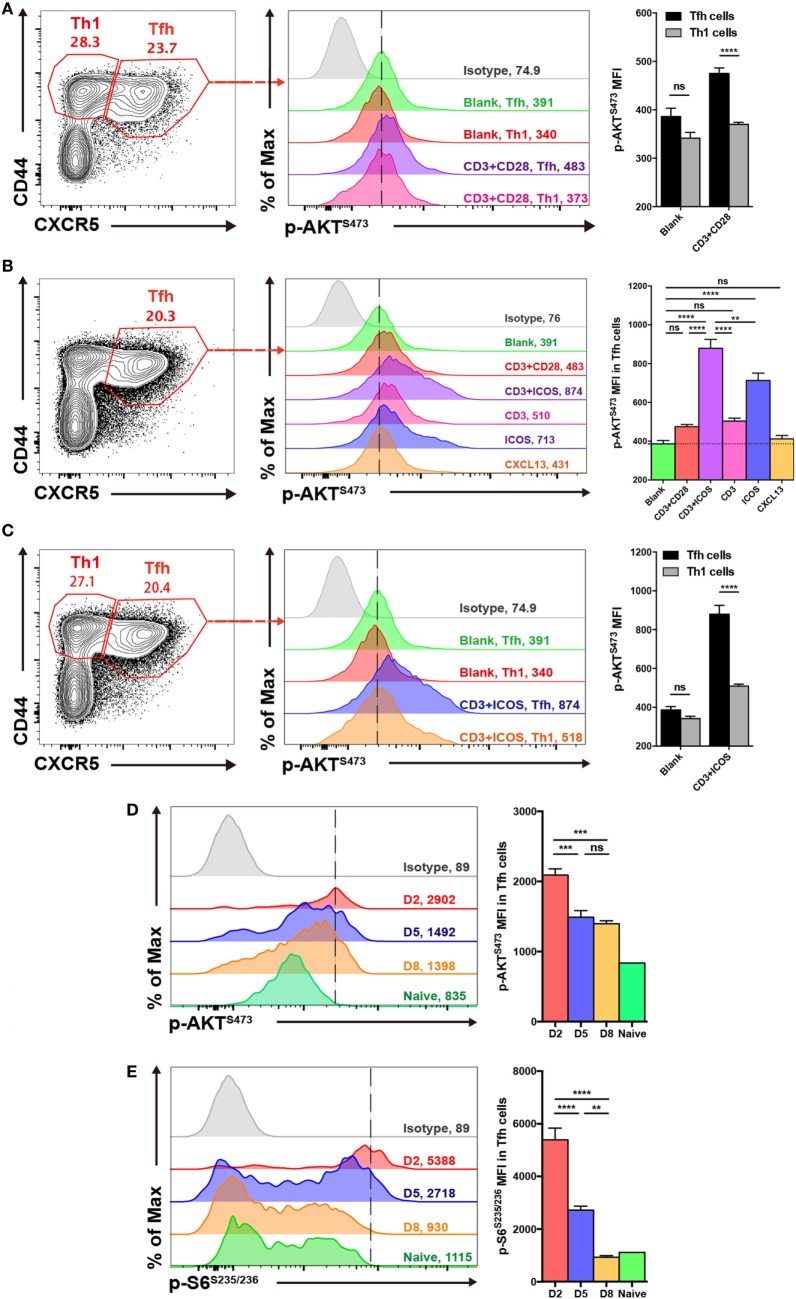
mTOR complex 2 activity is elevated in follicular helper CD4^+^ T (T_FH_) cells upon stimulation. **(A,C)** Comparison of p-AKT^S473^ mean fluorescence intensity between T_FH_ (CD44^hi^CXCR5^+^) and T_H_1 (CD44^hi^CXCR5^−^) cells from the spleen of wild-type C57BL/6J mice at day 8 after lymphocytic choriomeningitis virus infection. **(A)** T_FH_ and T_H_1 cells were cultured in medium without any stimulus (blank), or stimulated with anti-CD3 and anti-CD28 (CD3^+^CD28), and the level of p-AKT^S473^ was detected by flow cytometry (*n* = 5 mice per group). **(B)** T_FH_ cells were activated with different stimuli, and green, red, purple, pink, blue, orange, and gray solid histograms represent the blank, anti-CD3 and anti-CD28, anti-CD3 and anti-ICOS, anti-CD3, anti-ICOS, anti-CXCL13, and isotype control, respectively (*n* = 5 mice per group). **(C)** T_FH_ and T_H_1 cells were cultured in blank medium or stimulated with anti-CD3 and anti-ICOS (CD3^+^ICOS), and the level of p-AKT^S473^ was detected by flow cytometry (*n* = 5 mice per group). **(D,E)** Kinetics of mTOR activity in SMARTA T_FH_ cells at day 2, 5, and 8 after infection. **(D)** SMARTA T_FH_ cells were activated with anti-CD3, anti-ICOS, anti-CD28, and gp61-80 peptide, and then the level of p-AKT^S473^ was detected by flow cytometry (*n* = 5 mice per group). **(E)** SMARTA T_FH_ cells were activated with anti-CD3, anti-CD28, and gp61-80 peptide, and then the level of p-S6^S235/236^ was detected by flow cytometry (*n* = 5 mice per group). ns, not significant, **p < 0.01, ***p < 0.001, ****p < 0.0001 [unpaired two-tailed *t*-test **(A,C)**, one-way ANOVA with multiple comparisons **(B,D,E)**]. Data are representative of three **(A–C)** or two **(D,E)** independent experiments. Error bars are SEM **(A–C)**.

To evaluate the activity of mTORC2 signaling in T_FH_ and T_H_1 cells in the context of acute viral infection, we infected wild-type C57BL/6J mice with the Armstrong strain of LCMV and measured the level of AKT phosphorylation at Ser 473, which is an indicator of mTORC2 activity, between T_FH_ and T_H_1 cells in the spleen on day 8 post-infection. Flow cytometry data demonstrated that T_FH_ cells possessed higher mTORC2 activity than T_H_1 cells upon anti-CD3 plus anti-CD28 stimulation (Figure 1A).

Furthermore, to investigate which stimuli were mainly responsible for mTORC2 activation in T_FH_ cells, we stimulated splenocytes from wild-type C57BL/6J mice at day 8 of infection with different combinations, including anti-CD3 plus anti-CD28, anti-CD3 plus anti-ICOS, anti-CD3 only, anti-ICOS only, or CXCL13 only. We then compared mTORC2 activity in T_FH_ cells under these different stimulation conditions and observed that the combination of anti-CD3 plus anti-ICOS elicited the highest mTORC2 signaling (Figure 1B). Next, stimulation of T_FH_ cells with anti-ICOS alone induced high levels of mTORC2 activity, ranking second only to anti-CD3 plus anti-ICOS (Figure 1B). Moreover, T_FH_ cells stimulated with either anti-CD3 or anti-CD3 plus anti-CD28 displayed a certain level of mTORC2 activity, while CXCL13 failed to effectively activate mTORC2 (Figure 1B). These results indicated that ICOS and CD3 signaling were pivotal for mTORC2 signaling activation in T_FH_ cells, while CD28 and CXCL13 signaling might not be necessary. And then, we also compared mTORC2 activity between T_FH_ and T_H_1 cells upon anti-CD3 plus anti-ICOS stimulation. Consistently, T_FH_ cells also exhibited enhanced mTORC2 activity compared with T_H_1 cells (Figure 1C).

Next, we analyzed the kinetics of mTOR activity in T_FH_ cells at day 2, 5, and 8 post infection. To achieve this, we adoptively transferred naive SMARTA (CD45.1^+^) cells with transgenic TCR specific to LCMV glycoprotein I-A^b^ epitope (49) into naive C57BL/6J (CD45.2^+^) mice, which were infected with LCMV Armstrong strain on the following day. We found that mTORC2 activity was rapidly induced in early differentiated T_FH_ cells at 48 h post-infection and subsequently maintained at days 5 and 8 post-infection (Figure 1D). In contrast, mTORC1 was highly induced at the initiation phase of T_FH_ differentiation but dramatically declined to the baseline later on (Figure 1E). Taken together, these results demonstrated that T_FH_ cells possessed higher mTORC2 activity compared with T_H_1 cells. Additionally, ICOS and CD3 signaling might act as important upstream activators of mTORC2 signaling in T_FH_ cells.

### mTORC2 Is Intrinsically Required for Effector T_FH_ Cell Differentiation

Since mTORC2 activity was upregulated in T_FH_ cells, we speculated that mTORC2 might play a critical role in T_FH_ cell responses during acute viral infection. To test this hypothesis, we generated *Rictor*^fl/fl^*Cd4*-Cre mice by crossing *Rictor*^fl/fl^ mice with transgenic *Cd4*-*Cre* mice to conditionally delete *Rictor* alleles in CD4^+^ T cells. Next, we infected *Rictor*^fl/fl^*Cd4*-Cre mice (called “*Rictor^−/−^* mice” here) and their *Rictor*^fl/fl^ littermates (called “WT mice” here) with LCMV Armstrong and measured the level of *Rictor* mRNA copies and mTORC2 activity in CD4^+^ T cells. We found that *Rictor* mRNA expression in both T_FH_ and T_H_1 cells sorted from *Rictor^−/−^* mice was undetectable (Figure S1A in Supplementary Material). Additionally, mTORC2 activity failed to be induced in *Rictor^−/−^* CD4^+^ T cells upon stimulation (Figure S1B in Supplementary Material). However, the level of phosphorylated S6 at Ser 235/236, which is indicative of mTORC1 activity, was comparable between *Rictor^−/−^* and WT CD4^+^ T cells upon stimulation (Figure S1B in Supplementary Material). Therefore, mTORC2 signaling was adequately abrogated in CD4^+^ T cells from *Rictor^−/−^* mice.

**Figure 2:**
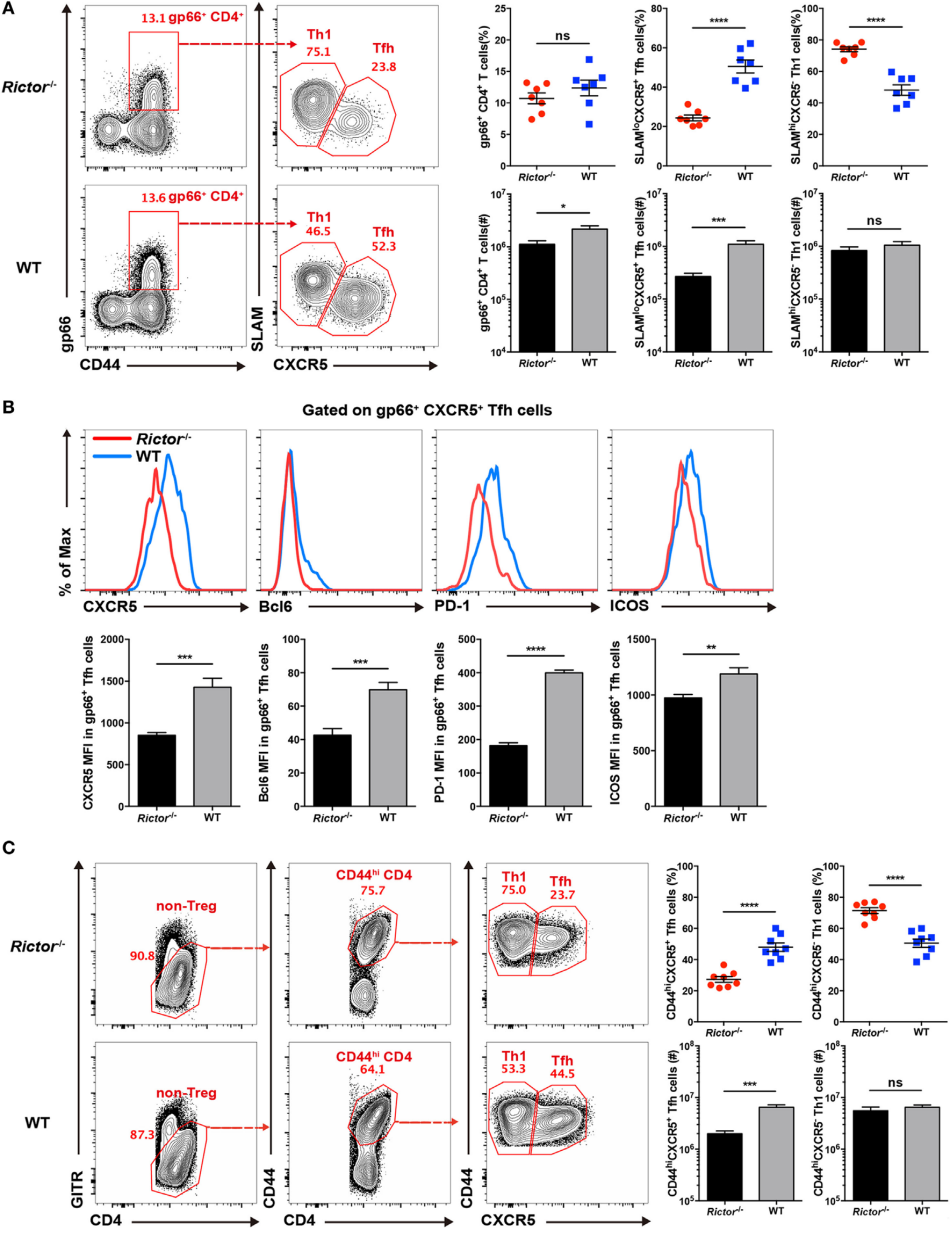

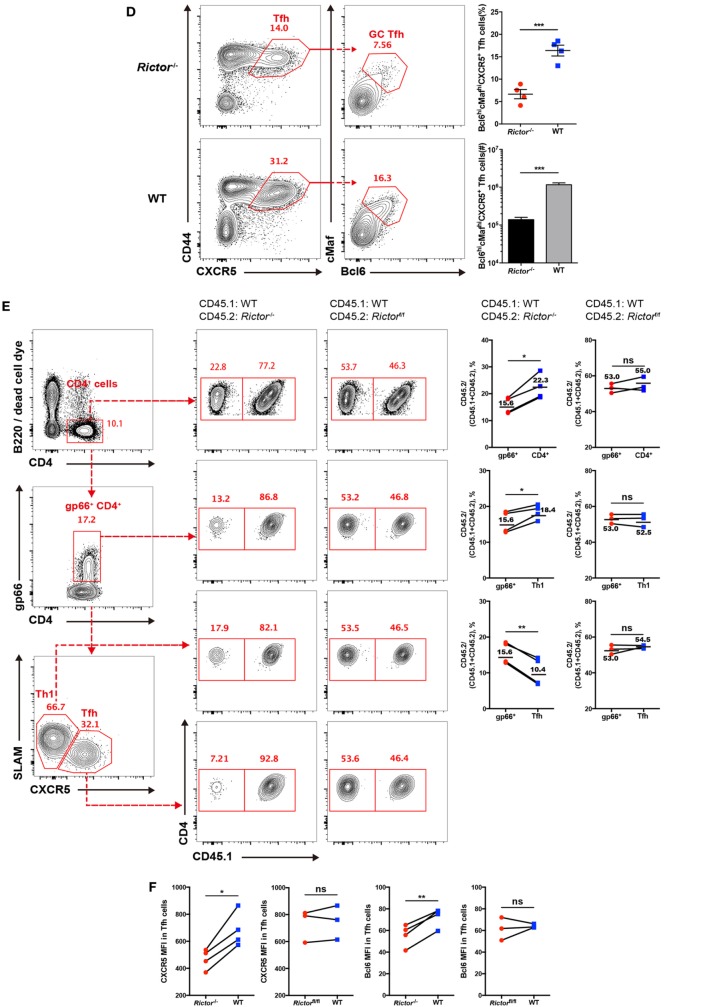
mTOR complex 2 is critical for follicular helper CD4^+^ T (T_FH_) differentiation during acute viral infection. **(A–D)** *Cd4-*Cre*Rictor*^fl/fl^ mice (*Rictor*^−/−^) and littermates *Rictor*^fl/fl^ (WT) mice were infected with lymphocytic choriomeningitis virus (LCMV) Armstrong, and CD4^+^ T cell responses were assessed in spleen at day 8 after infection. **(A)** Flow cytometry of T_FH_ (SLAM^lo^CXCR5^+^) and T_H_1 (SLAM^hi^CXCR5^−^) cells among gp66 tetramer-positive CD4^+^ T (gp66^+^ CD4^+^) cells of *Rictor*^−/−^ (top) and WT mice (bottom) (left), and summary of the frequency (top) and cell number (bottom) (right) (*n* = 8 mice per group). **(B)** Representative flow cytometry plots showing the expression levels of CXCR5, Bcl-6, PD-1, and ICOS on tetramer-positive T_FH_ cells as in **(A)** from *Rictor^−/−^* and WT mice (top), and summary of the mean fluorescence intensity (bottom) (*n* = 8 mice per group). **(C)** Flow cytometry of bulk activated T_FH_ (GITR^lo^CD44^hi^CXCR5^+^) and T_H_1 (GITR^lo^CD44^hi^CXCR5^−^) cells of *Rictor^−/−^* (top) and WT mice (bottom) (left), and summary of the frequency (top) and cell number (bottom) (right) (*n* = 8 mice per group). **(D)** Flow cytometry of germinal center T_FH_ cells (Bcl6^hi^cMaf^hi^) among bulk activated T_FH_ cells (GITR^lo^CD44^hi^CXCR5^+^) of *Rictor^−/−^* (top) and WT mice (bottom) (left), and summary of the frequency (top) and cell number (bottom) (right) (*n* = 4–5 mice per group). **(E,F)** Flow cytometry of CD4^+^ T cell responses in the spleen of KO versus WT bone marrow (BM) chimeras at 8 days after LCMV infection. KO BM chimeras were generated with a mixture of BM cells from *Rictor^−/−^* (CD45.2^+^) and C57BL/6J (WT) (CD45.1^+^) mice, and WT BM chimeras were reconstituted with a mixture of BM cells from *Rictor*^fl/fl^ (CD45.2^+^) and C57BL/6J (WT) (CD45.1^+^) mice. **(E)** Representative flow cytometry plots showing competitive contributions by CD45.2^+^ cells to the total CD4^+^, gp66^+^ CD4^+^, gp66^+^ T_FH_, and T_H_1 cell population in KO and WT BM chimeras (left). Comparison of the contribution of CD45.2^+^ cells to gp66^+^ CD4^+^ versus total CD4^+^, gp66^+^ CD4^+^ versus gp66^+^ T_FH_, and gp66^+^ CD4^+^ versus gp66^+^ T_H_1, respectively (right) (*n* = 4 mice per group). **(F)** Expression of CXCR5 and Bcl-6 in KO and WT BM chimeras (*n* = 4 mice per group). ns, not significant, *p < 0.05, **p < 0.01, ***p < 0.001, ****p < 0.0001 [unpaired **(A–D)** or paired **(E,F)** two-tailed *t*-test]. Data are representative of four **(A–D)** or two **(E,F)** independent experiments. Error bars are SEM **(A–F)**.

Next, we analyzed CD4^+^ T cell responses at day 8 after LCMV infection, and we found a reduced cell number of total virus-activated CD44^hi^CD4^+^ T cells in *Rictor^−/−^* mice than control mice (Figure S1C in Supplementary Material) and a similar frequency but lower number of I-A^b^ restricted LCMV-gp66 epitope-specific CD4^+^ T cells (Figure 2A). Notably, both the frequency and cell number of tetramer-positive SLAM^lo^CXCR5^+^ T_FH_ cells were greatly decreased in *Rictor^−/−^* mice, whereas the tetramer-positive SLAM^hi^CXCR5^−^ T_H_1 cells showed an increased frequency and similar cell number (Figure 2A). Thus, the reduction of total CD44^hi^CD4^+^ T cells and tetramer-positive CD4^+^ T cells were mainly attributable to a decrease in T_FH_ but not T_H_1 cells, which was in agreement with a previous study showing that mTORC2 was dispensable for T_H_1 differentiation (32). Moreover, we also measured the expression of CXCR5, Bcl-6, PD-1, and ICOS in tetramer-positive T_FH_ cells and found that all of these T_FH_ cell-associated molecules were downregulated in *Rictor^−/−^* mice compared with the WT control (Figure 2B). In addition to tetramer-positive CD4^+^ T cells, we also analyzed the responses of bulk activated CD4^+^ T cell and observed similar phenotypes in *Rictor^−/−^* mice (Figure 2C; Figure S1D in Supplementary Material). Notably, we observed an approximately 3-fold lower frequency and 10-fold lower cell numbers of Bcl6^hi^cMaf^hi^ GC T_FH_ cells in *Rictor^−/−^* compared with WT mice (Figure 2D), suggesting an impairment of T_FH_ cell maturation in the absence of mTORC2 signaling. These results showed that mTORC2 signaling was essential for T_FH_ differentiation, but dispensable for T_H_1 differentiation.

However, there was a potential concern that CD8^+^ T cells in *Rictor^−/−^* mice were also mTORC2 signaling deficient, which might impact the viral clearance rate and further confound T_FH_ responses. In addition, the impaired T_FH_ cell differentiation in *Rictor^−/−^* mice would result in poorer GC B cell responses, which in turn might negatively influence T_FH_ cell responses as a feedback loop. To more precisely assess the role of mTORC2 signaling in T_FH_ cell responses, we established bone marrow (BM) chimeras by transferring cell mixtures of BM cells derived from *Rictor^−/−^* mice (CD45.2^+^) (40%) and C57BL/6J mice (CD45.1^+^) (60%) into irradiated C57BL/6J recipients (CD45.1^+^). Control groups were also generated by transferring BM cell mixtures derived from *Rictor*^fl/fl^ mice (CD45.2^+^) (40%) and C57BL/6J mice (CD45.1^+^) (60%). After 8 weeks of reconstitution, we infected these BM chimeras with LCMV Armstrong and analyzed T_FH_ responses at day 8 after infection. First, we gated on the total CD4^+^ T cell population, CD4^+^gp66^+^ population, and gp66^+^ T_FH_ and T_H_1 populations, and then we compared the contribution of CD45.2^+^ cells derived from *Rictor^−/−^* or *Rictor*^fl/fl^ mice among these populations (Figure 2E). We found that CD45.2^+^ CD4^+^ T cells originating from *Rictor^−/−^* mice accounted for approximately 22.3% of the total CD4^+^ T cell population; however, CD45.2^+^ gp66^+^ cells of Rictor*^−/−^* origin accounted for approximately 15.6% of the total gp66^+^ T cell subset (Figure 2E). Moreover, *Rictor*-null CD45.2^+^ T_H_1 cells contributed approximately 18.4% of the total T_H_1 cells, while CD45.2^+^ T_FH_ cell derived from *Rictor^−/−^* mice contributed only approximately 10.4% of the total T_FH_ cells (Figure 2E). In control BM chimeras, however, CD45.2^+^ cells of *Rictor*^fl/fl^ origin exhibited a stable contribution from 52.53 to 55% among the total CD4^+^ T, CD4^+^gp66^+^, gp66^+^ T_H_1, and gp66^+^ T_FH_ populations (Figure 2E). Additionally, the expression levels of CXCR5 and Bcl-6 were reduced in *Rictor^−/−^* T_FH_ cells, but not *Rictor*^fl/fl^ T_FH_ cells, compared with the control group (Figure 2F). The results from the BM chimera model collectively illustrated that T_FH_ cell differentiation was specifically dampened in the absence of mTORC2 signaling, and more importantly, mTORC2 was required for T_FH_ differentiation in a cell-autonomous manner.

In addition to LCMV infection, we also analyzed T_FH_ responses in different immunization models by infecting *Rictor^−/−^* and WT mice with *Listeria monocytogenes* expressing a CD4^+^ T cell epitope derived from LCMV gp61-80 (LM-GP61). Phenotypes were observed on day 8 post-infection. In agreement with the results from the LCMV infection, both gp66^+^ and activated bulk T_FH_ cells from *Rictor^−/−^* mice exhibited decreased frequencies and cell numbers compared with *Rictor*^fl/fl^ littermates (Figure S2A in Supplementary Material). Additionally, similar results were obtained using the 4-hydroxy-3-NP-OVA immunization model (Figure S2B in Supplementary Material). Thus, mTORC2 signaling also played a critical role in T_FH_ cell responses in both bacterial and protein immunization models.

To exclude the possibility that impaired T_FH_ differentiation in *Rictor^−/−^* mice was due to defects in CD4^+^ T cells during T cell development or the naïve state, we assessed the frequencies and cell numbers of single CD4^+^ T cells, single CD8^+^ T cells, double-positive, and double-negative T cells in thymus and found no significant differences in these parameters except a slight increase in cell number of double-negative T cells in *Rictor^−/−^* mice (Figure S3A in Supplementary Material). We then compared total CD4^+^ T cell, CD4^+^CD62L^hi^ T cell, and CD4^+^CD44^hi^ T cell populations in spleens from *Rictor^−/−^* or WT mice in the naïve state and observed a slightly lower frequency but comparable cell number of total CD4^+^ T cells in the *Rictor^−/−^* group (Figure S3B in Supplementary Material). In addition, the other two populations both displayed similar cell frequencies and numbers between *Rictor^−/−^* and WT mice (Figure S3B in Supplementary Material). Therefore, we showed that the decrease in the T_FH_ cell population in *Rictor^−/−^* mice was not due to defects in T cell development and homeostasis. Notably, our previous data have shown that the loss of mTORC2 signaling does not alter the generation of T_FR_ cells (39), which indicates that the reduction of the T_FH_ cell population in *Rictor^−/−^* mice was probably not due to increased suppression by T_FR_ cells. Therefore, these data demonstrated that intact mTORC2 signaling was essential for effector T_FH_ cell differentiation in viral infection, bacterial infection, and protein immunization models.

### Intact mTORC2 Signaling Is Dispensable for T_FH_ Early Induction, but Critical for Late Maturation During T_FH_ Differentiation

**Figure 3:**
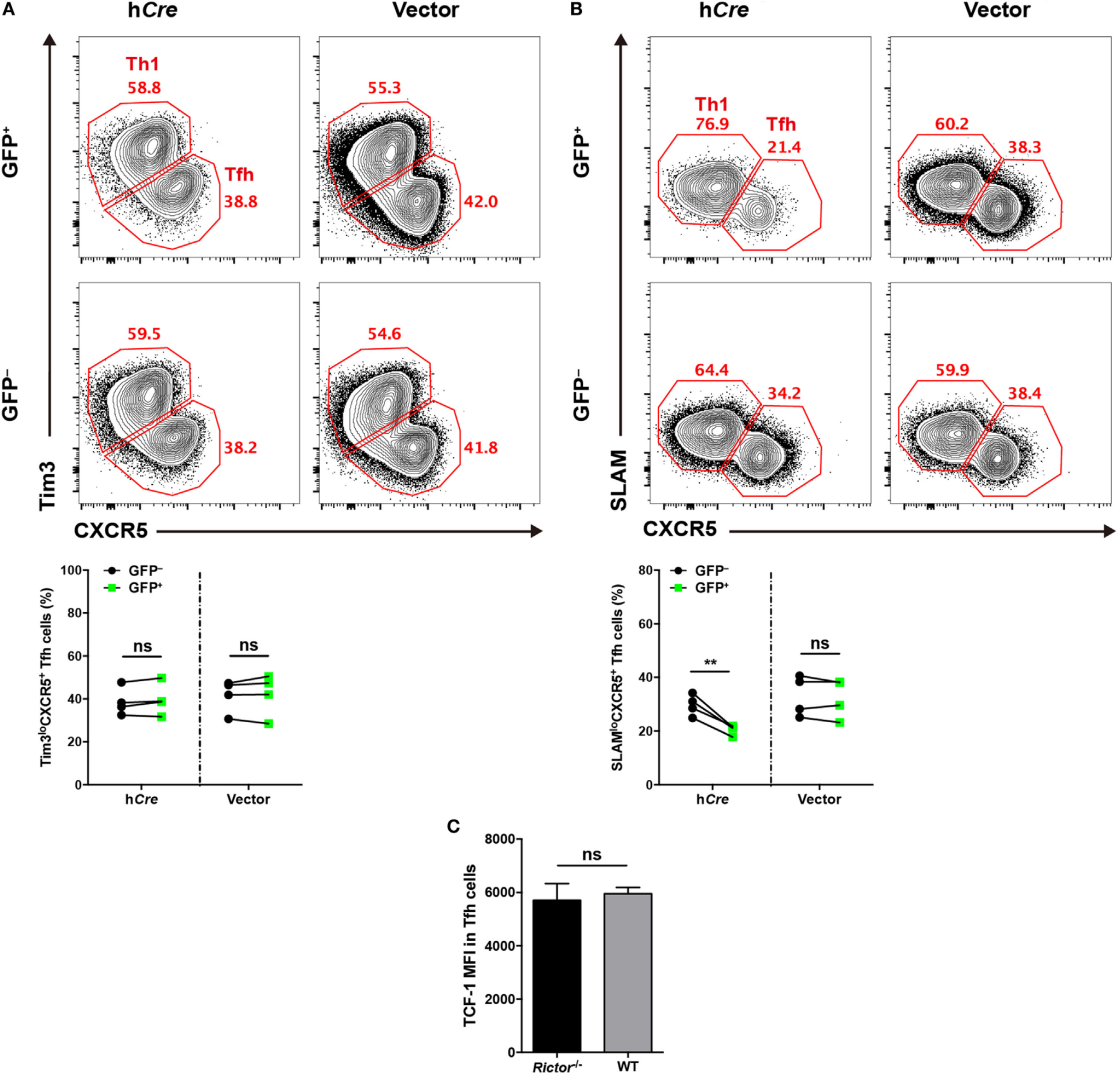
mTOR complex 2 is selectively required for late follicular helper CD4^+^ T (T_FH_) differentiation, but not early induction. **(A,B)** *Rictor*^fl/fl^-SMARTA cells were transduced with retrovirus expressing humanized-*Cre* (h*Cre*) or empty vector (vector) and then transferred cells into recipients (CD45.2^+^) and subsequently infected with lymphocytic choriomeningitis virus (LCMV). Flow cytometry of T_FH_ differentiation in *Rictor*^fl/fl^-SMARTA cells transduced (GFP^+^) with h*Cre* or vector, or non-transduced (GFP^−^) at day 3 **(A)** and 8 **(B)** after infection (*n* = 4 mice per group). **(C)** T cell factor-1 expression in early differentiated T_FH_ cells at day 3 after infection. *Rictor^−/−^* or WT SMARTA (CD45.1^+^) cells were adoptively transferred into naive C57BL/6J (CD45.2^+^) mice, following intravenously infection with 2 × 10^6^ plaque-forming units of LCMV and analyzed at day 3 after infection (*n* = 4–6 mice per group). ns, not significant, **p < 0.01 [paired two-tailed *t*-test **(A,B)**, unpaired two-tailed *t*-test **(C)**]. Data are representative of two **(A–C)** independent experiments. Error bars are SEM **(A–C)**.

To distinguish the role of mTORC2 in early commitment induction or late maturation during T_FH_ differentiation, respectively, we crossed *Rictor*^fl/fl^ mice with SMARTA mice to generate *Rictor*^fl/fl^-SMARTA mice, which enabled us to delete *Rictor* in SMARTA cells by transducing the humanized - *Cre* (call “h*Cre*” here) retroviral expression vector in activated SMARTA cells. To achieve this goal, we transduced activated SMARTA cells (CD45.1^+^) with h*Cre* expressing vector or control empty vector and then adoptively transferred them into WT C57BL/6J recipient mice (CD45.2^+^), which were subsequently infected with LCMV Armstrong. First, we assessed early T_FH_ differentiation at day 3 after infection, and interestingly, we found that h*Cre*-transduced (GFP^+^) SMARTA cells with *Rictor* knocked out showed a similar frequency of Tim3^lo^CXCR5^+^ T_FH_ cells to empty vector-transduced (GFP^+^) and non-transduced (GFP^−^) SMARTA cells (Figure 3A). This result suggested that early differentiated T_FH_ cells were dispensable for mTORC2 signaling.

Next, we estimated late T_FH_ differentiation at day 8 after infection in comparison to day 3, and we observed a lower abundance of SLAM^lo^CXCR5^+^ T_FH_ cells differentiating from h*Cre*-transduced SMARTA cells relative to their non-transduced compartments (Figure 3B). However, SMARTA cells transduced with empty vector expressing only GFP showed an equal frequency of T_FH_ cells compared with non-transduced ones (Figure 3B).

Additionally, we generated *Rictor*^fl/fl^*Cd4*-Cre-SMARTA mice (called “*Rictor^–/–^* SMARTA mice” here) by crossing *Rictor*^fl/fl^ *Cd4*-Cre mice with SMARTA mice to specifically delete *Rictor* in SMARTA cells. Then, we adoptively transferred *Rictor^−/−^* or WT SMARTA cells (CD45.1^+^) into C57BL/6J mice (CD45.2^+^) and subsequently infected these recipients with LCMV. At day 3 post-infection, we compared the expression of TCF-1 in early differentiated T_FH_ cells from *Rictor^−/−^* and WT SMARTA cells, respectively. It turned out that TCF-1 expression level was comparable between these T_FH_ cells, indicating that TCF-1 induction was independent of mTORC2 activity at the early T_FH_ differentiation (Figure 3C). Taken together, these data indicated that mTORC2 signaling was not required for early induction of T_FH_ cells, whereas it played a pivotal role in late maturation of T_FH_ cells.

### Defective Responses of mTORC2-Deficient T_FH_ Cells Lead to Impaired Humoral Immunity

**Figure 4:**
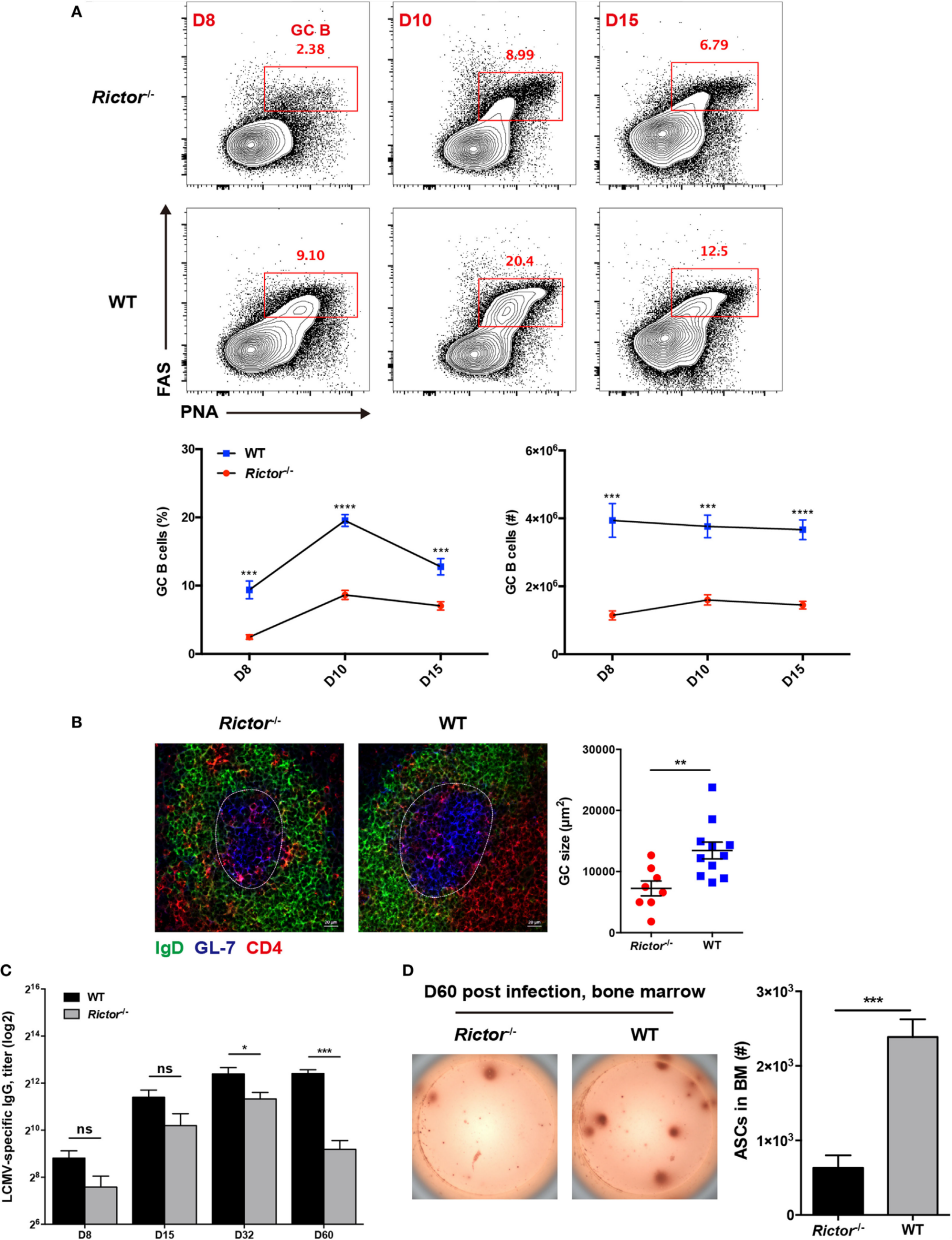
Impaired follicular helper CD4^+^ T (T_FH_) differentiation leads to repressed humoral immunity in *Rictor^−/−^* mice. **(A)** Representative flow cytometry plots showing germinal center (GC) B (peanut agglutinin^hi^FAS^hi^) cells in *Rictor^−/−^* versus WT mice at day 8, 10, and 15 after lymphocytic choriomeningitis virus (LCMV) infection (top). Summary of GC B cell frequency and cell number (bottom) (*n* = 5–8 mice per group). **(B)** Confocal microscopy analysis of GC histology in spleen sections from *Rictor^−/−^* and WT mice at day 8 after LCMV infection (green: IgD, blue: GL-7, red: CD4) (Scale bar: 100 µm) (left). Quantification of GC size (right) (*n* = 3 mice per group). **(C)** Titers of LCMV-specific immunoglobulin G in serum from *Rictor^−/−^* and WT mice at day 8, 15, 32, and 60 after LCMV infection were detected by enzyme-linked immunosorbent assay (*n* = 5–7 mice per group). **(D)** Numbers of LCMV-specific antibody-secreting cells (ASCs) in bone marrow from *Rictor^−/−^* and WT mice at day 60 after infection, calculated by the enzyme-linked immunospot assay (*n* = 5 mice per group). ns, not significant, *p < 0.05, **p < 0.01, ***p < 0.001, ****p < 0.0001 [unpaired two-tailed *t*-test **(A–D)**]. Data are representative of three **(A)** or two **(B–D)** independent experiments. Error bars are SEM **(A–D)**.

Follicular helper CD4^+^ T cells provide help to cognate B cells to initiate GC reactions and promote the further differentiation of GC B cells into memory B cells and long-lived plasma cells (7). Given the crucial role of T_FH_ cell in B cell responses, the defective T_FH_ differentiation in the absence of mTORC2 appears to have a negative impact on B cell responses. To validate this hypothesis, we first compared the kinetics of GC B cells between *Rictor* null and WT mice at day 8, 10, and 15 after infection. As expected, we observed a great decrease in the frequencies and cell numbers of GC B cells, as defined by high expression of Fas (CD95) and peanut agglutinin (PNA) (Fas^hi^PNA^hi^) in *Rictor^−/−^* mice at all time points (Figure 4A). Likewise, we also observed similar alterations of GC B cells in the *Listeria*-gp66 and NP-OVA protein immunization model at day 8 after immunization (Figures S4A, B in Supplementary Material). In addition to the decrease in GC B cells, GCs in spleens from *Rictor^−/−^* mice were smaller than those in WT mice, and fewer mTORC2-deficient T_FH_ cells could enter and localize within GCs and B cell follicles than WT control (Figure 4B).

Next, we measured LCMV-specific IgG titers in sera at multiple time points after LCMV infection and found that IgG titers were not significantly reduced from day 8 to day 32 in *Rictor-*deficient compared to control mice; however, they were remarkably downregulated at day 60, indicative of impaired long-term antibody responses (Figure 4C). Consistently, GC-derived LCMV-specific ASCs in the BM were found to be greatly diminished in *Rictor^−/−^* compared with WT mice at day 60 post-infection (Figure 4D). Taken together, these data led to the notion that *Rictor*-deficient mice failed to sustain effective and long-term humoral immunity, which resulted from impaired T_FH_ differentiation.

### mTORC2 Regulates Migratory and Functional Properties of Differentiated T_FH_ Cells

**Figure 5:**
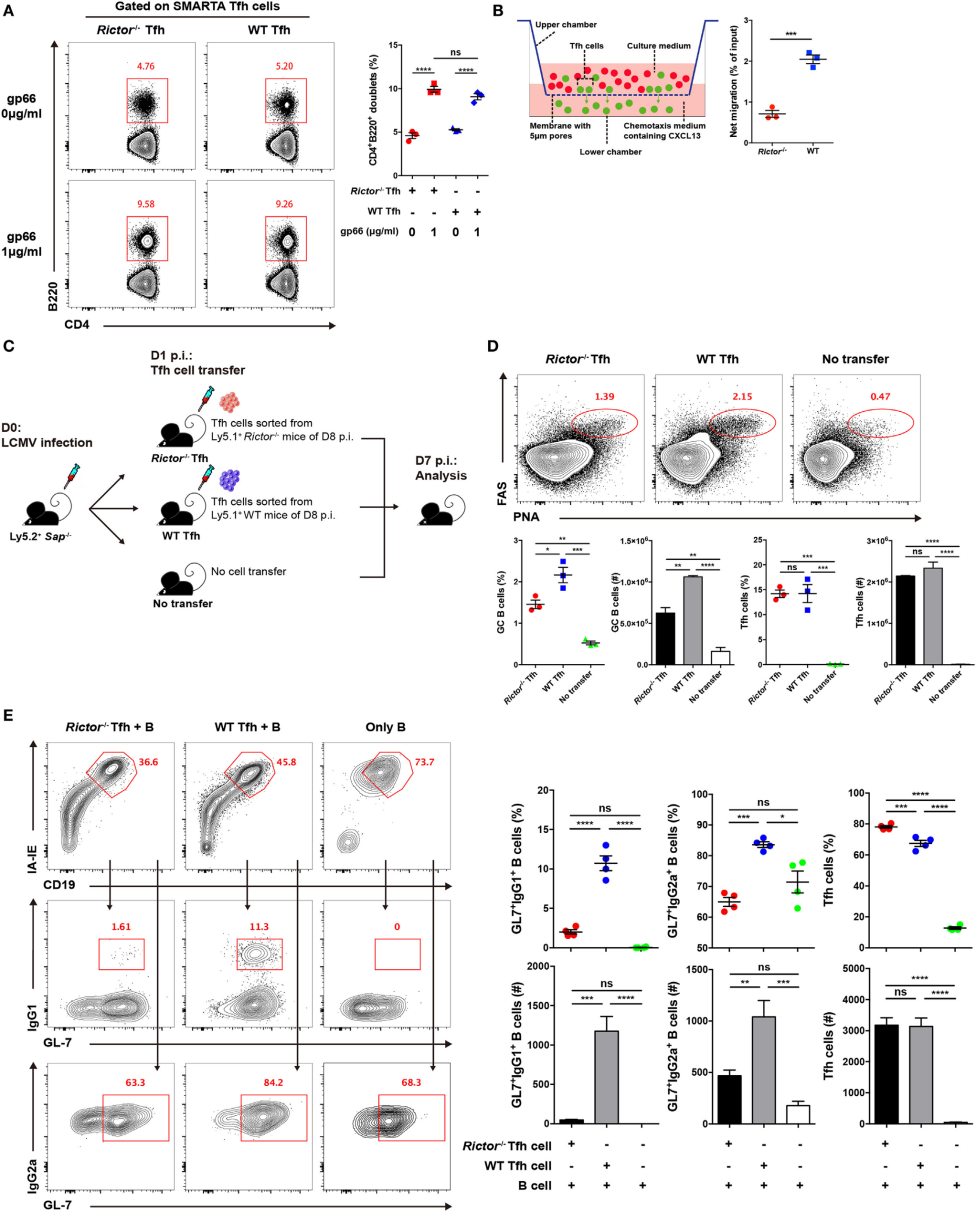
mTOR complex 2 plays an essential role in regulating the migratory ability and effector function of follicular helper CD4^+^ T (T_FH_) cells. **(A)** *Rictor^−/−^* or WT SMARTA T_FH_ cells were incubated with LPS-activated B cells pulsed with cognate gp66-peptide or unpulsed (left). The frequency of T cell–B cell conjugates among total SMARTA T_FH_ cells was quantified by flow cytometry (right) (*n* = 3 samples per group). **(B)** Working model of the chemotaxis transwell assay for T_FH_ cells (left). The absolute number of migrated T_FH_ cells was quantified by flow cytometry, and the net migration efficiency (% of input) was calculated (right) (*n* = 3 samples per group). **(C)** Experimental setup of the *in vivo* T_FH_ function assay. **(D)** Representative flow cytometry results of the *in vivo* T_FH_ function assay (top), *Sap^−/−^* mice receiving exogenous T_FH_ cells from *Rictor^−/−^* (left) and WT mice (middle) or negative control without cell transfer (right) (top). Summary of frequency and total number of germinal center B cell and exogenous transferred T_FH_ cells in *Sap^−/−^* host mice (bottom) (*n* = 3 mice per group). **(E)** *In vitro* T_FH_ function assay assessed at day 4 after culture. Flow cytometry of immunoglobulin G (IgG1)^+^GL-7^+^ and IgG2a^+^GL-7^+^ GC-like B cells cultured with *Rictor^−/−^* or WT T_FH_ cells or in the absence of T_FH_ cells (left). Quantification of the frequencies and total numbers of IgG1^+^GL-7^+^, IgG2a^+^GL-7^+^ GC-like B cells and T_FH_ cells in culture (right) (*n* = 4 samples per group). ns, not significant, *p < 0.05, **p < 0.01, ***p < 0.001, ****p < 0.0001 [unpaired two-tailed *t*-test **(B)**, one-way ANOVA with multiple comparisons **(A,D,E)**]. Data are representative of two **(A–E)** independent experiments. Error bars are SEM **(A,B,D,E)**.

Next, we further investigated how mTORC2 regulated T_FH_ cell differentiation and consequently affected humoral immunity. Cognate interactions between T_FH_ and B cells are essential for priming and maintenance of GC responses (50–52), differentiation of memory B cells and long-lived plasma cells (21, 53, 54), and complete differentiation of T_FH_ cells (5, 9, 55). We investigated whether mTORC2 signaling was responsible for the formation of T cell–B cell junctions. To achieve this goal, we constituted SMARTA chimeras by adoptively transferring *Rictor*^fl/fl^*Cd4*-Cre-SMARTA (called “*Rictor^–/–^* SMARTA” here) or WT SMARTA cells (CD45.1^+^) into C57BL/6J mice (CD45.2^+^) and subsequently infecting these recipients with LCMV. At day 8 after infection, SMARTA T_FH_ cells were sorted from *Rictor^−/−^* and WT SMARTA chimeras and cultured with LPS-activated B cells pulsed or not with gp66 peptide. The T cell–B cell conjugates, which were identified as CD4^+^B220^+^ doublets, were quantified by flow cytometry. We found that the frequency of T cell–B cell conjugates was substantially higher in gp66-pulsed groups than unpulsed negative controls (Figure 5A). Of note, a similar frequency of T cell–B cell conjugates was observed between the *Rictor^−/−^* and WT group in the presence of gp66 (Figure 5A), and similar results were observed using gp66 tetramer^+^ T_FH_ cells sorted from *Rictor^−/−^* and WT mice (Figure S5A in Supplementary Material). These data indicated that the cell adhesion between T_FH_ and B cells was independent of mTORC2 signaling.

High expression of CXCR5 in T_FH_ cells facilitates the response of these cells to the chemokine CXCL13 and migration toward B cell follicles (1–6), where they can engage cognate B cells. Next, to determine whether mTORC2 regulates T_FH_ cell responses by altering its migratory pattern, we conducted a transwell migration assay to analyze the mobility of the T_FH_ cells and ability to directionally respond to CXCL13. We added equal numbers of WT and *Rictor^−/−^* T_FH_ cells in the upper chamber of the transwell plate and added medium supplemented with or without CXCL13 in the lower chamber. After 3 h of migration, we found that the number of migrated *Rictor^−/−^* T_FH_ cells was largely decreased compared with the WT controls (Figure 5B), suggesting an impairment of the migratory potential of T_FH_ cells lacking mTORC2 signaling in response to CXCL13, which might result in reduced colocalization of T_FH_ and GC B cells and, therefore, constrained T_FH_ differentiation and GC responses.

Finally, we assessed the impact of mTORC2 signaling on the capacity of T_FH_ cells to provide the helper signals for B cell survival and activation. To evaluate the helper function of T_FH_ cell *in vivo*, we sorted fully differentiated T_FH_ cells from day 8-infected *Rictor^−/−^* or WT mice (CD45.1^+^) and transferred equal number of these cells into *Sh2d1a^−/−^* (called *Sap^−/−^* here) (CD45.2^+^) recipients at day 1 of infection (Figure 5C). At day 7 after infection of recipients (6 days after cell transfer), we observed a distinct Fas^hi^PNA^hi^ GC B cell population in mice that received exogenous T_FH_ cells from *Rictor^−/−^* or WT donor, while mice without cell transfer displayed barely detectable GC B cells (Figure 5D). Additionally, the number of transferred T_FH_ cells was minimally altered (Figure 5D). Importantly, we found that mice that received *Rictor^−/−^* T_FH_ cells exhibited an approximately 2-fold lower frequency and number of GC B cells than control mice (Figure 5D), which suggested a disrupted effector function of *Rictor*-deficient T_FH_ cells.

To further confirm this point, we utilized another approach to assess T_FH_ function *in vitro*. We sorted fully differentiated T_FH_ cells from *Rictor^−/−^* or WT mice and B cells from C57BL/6J mice at day 8 after infection of LCMV and then cultured T_FH_ cells with B cells for 4 days in the presence of anti-CD3 and anti-IgM. Next, we found that the B cells cultured with WT T_FH_ cells formed a distinct population of germinal center-like B cells determined by high expression of IgG1 and GL-7 (IgG1^hi^GL-7^hi^) or IgG2a and GL-7 (IgG2a^hi^GL-7^hi^), whereas B cells cultured with *Rictor^−/−^* T_FH_ cells exhibited a much lower frequency and cell number of activated B cells than that in the WT group (Figure 5E). Moreover, the numbers of *Rictor^−/−^* and WT T_FH_ cells were comparable after 4 days of culture (Figure 5E). These data suggested that mTORC2 signaling was essential for the effector function of already differentiated T_FH_ cells to promote B cell differentiation. Therefore, activated B cell responses critically depended on the presence of cognate T_FH_ cells with competent mTORC2 signaling.

### mTORC2 Plays an Essential Role in T_FH_ Cell Lineage Identity

**Figure 6:**
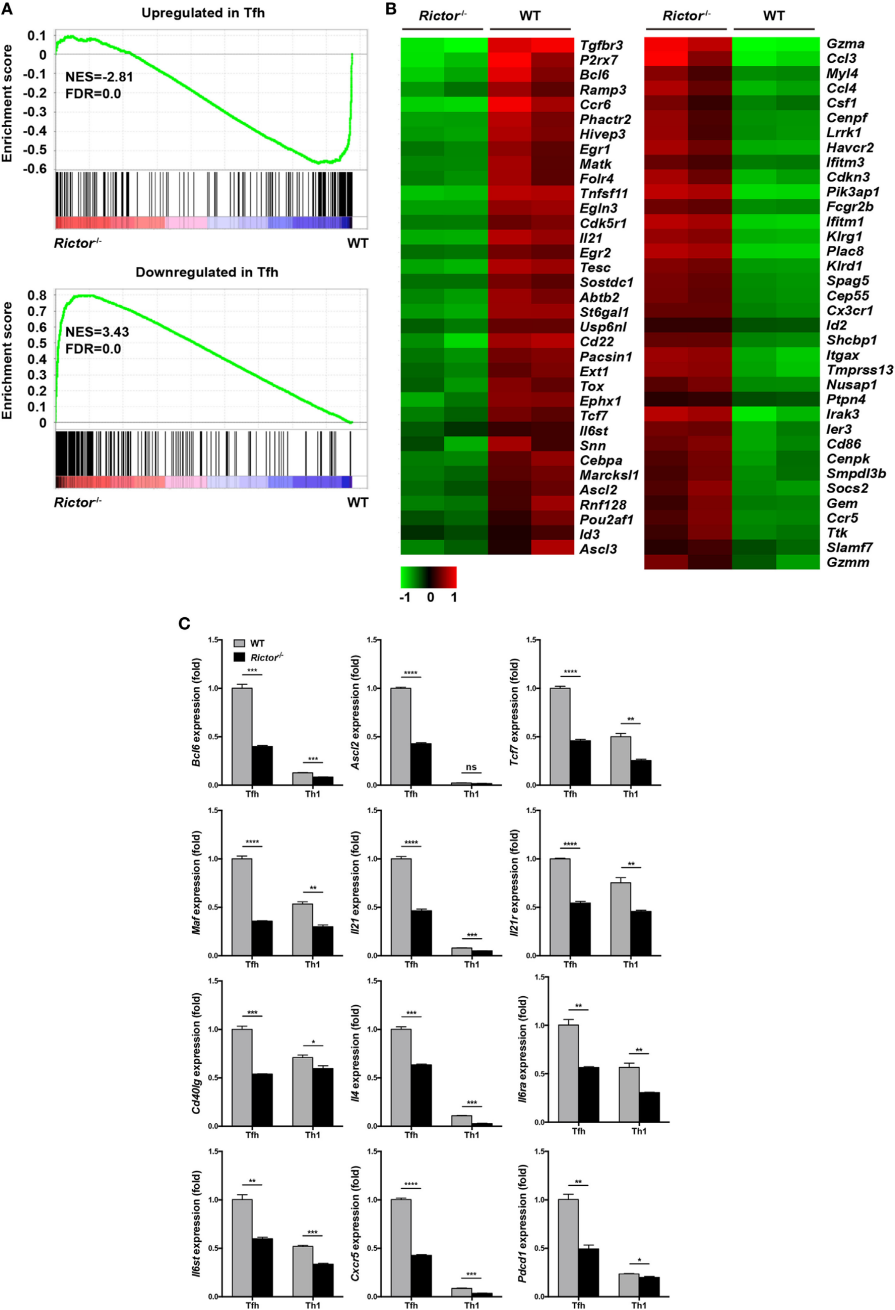
mTOR complex 2 transcriptionally regulates signature programming of follicular helper CD4^+^ T (T_FH_) cells. **(A)** Gene-set-enrichment analysis of gene signatures that were upregulated (top) or downregulated (bottom) in T_FH_ cells relative to their expression in non-T_FH_ cells, T_FH_ cells were sorted from *Rictor^−/−^* and WT mice at day 8 after infection with lymphocytic choriomeningitis virus (GEO accession codes GSE21379 and GSE21381). **(B)** Heat-map representation of the expression of the top 71 genes selected from **(A)**. Red, high gene expression; green, low gene expression. **(C)** RT-quantitative polymerase chain reaction of selected 12 genes from the microarray data (see Table S3 in Supplementary Material) normalized to the expression level in WT T_FH_ cells (*n* = 3 samples per group). ns, not significant, *p < 0.05, **p < 0.01, ***p < 0.001, ****p < 0.0001 [unpaired two-tailed *t*-test **(C)**]. Data are from one experiment with two biological replicates pooled from six *Rictor^−/−^* mice and four WT mice per group **(A,B)** or are representative of two independent experiments **(C)**. Error bars are SEM **(C)**.

To understand the molecular mechanisms by which mTORC2 transcriptionally regulates T_FH_ differentiation and function, we sorted T_FH_ and T_H_1 cells from *Rictor^−/−^* and WT mice at day 8 after infection and performed gene expression microarray analysis. Microarray analysis showed that 561 genes were upregulated and 201 genes were downregulated in *Rictor*-deficient T_FH_ cells relative to their WT counterparts (Table S3 in Supplementary Material). To further analyze the transcriptomic alterations in *Rictor^−/−^* T_FH_ cells, we next selected sets of genes from published datasets (GEO accession codes GSE21379 and GSE21381) that are upregulated and downregulated in T_FH_ cells compared with non-T_FH_ cells (56), for GSEA. GSEA analysis illustrated that the genes related to the T_FH_ cell signature (upregulated in T_FH_ cells) were more enriched in WT T_FH_ cells, but not *Rictor^−/−^* T_FH_ cells (Figure 6A). By contrast, *Rictor^−/−^* T_FH_ cells showed enrichment for the gene set associated with the non-T_FH_ lineage (downregulated in T_FH_ cells) (Figure 6A). We then assessed 71 genes from the GSEA results and observed a distinct gene expression profile between *Rictor^−/−^* T_FH_ cells and WT T_FH_ cells (Figure 6B). These results suggested that mTORC2 signaling deficiency resulted in disruption of T_FH_ lineage specification.

Next, we selected several genes that are closely involved in T_FH_ differentiation and function from the microarray results and confirmed their alterations by quantitative polymerase chain reaction (qPCR) (Figure 6C). We noted that the expression of *Bcl6, Ascl2, and Tcf7*, which encode key transcription factors in T_FH_ cells, were remarkably downregulated in *Rictor*-deficient T_FH_ compared with WT T_FH_ cells (Figure 6C). Moreover, we also found that *Rictor^−/−^* T_FH_ cells exhibited a lower mRNA abundance of *Maf*, a c-Maf encoding gene, which induces the expression of IL-21 in T_FH_ cells to support GC development (57, 58) (Figure 6C). Accordingly, both the levels of *Il21* and *Il21r* were downregulated in *Rictor*-null T_FH_ compared with WT cells (Figure 6C). Similarly, the expression levels of *Cd40lg* and *Il4*, encoding CD40L and IL-4 to promote B cell differentiation, were lower in *Rictor^−/−^* T_FH_ cells (Figure 6C). Additionally, the expression levels of both *Il6ra* and *Il6st*, which encode the IL-6R and gp130 receptors for IL-6, respectively, and are essential for instructing early T_FH_ differentiation, were decreased in *Rictor^−/−^* T_FH_ cells (13, 59) (Figure 6C). In addition, we observed a reduction of *Cxcr5* and *Pdcd1*, which respectively encode the T_FH_ distinguishing markers CXCR5 and PD-1 in *Rictor^−/−^* T_FH_ cells (Figure 6C). These data suggested that mTORC2 played a critical role in maintaining both T_FH_ lineage identity and functionality.

## Discussion

In this study, we focused on dissecting the role of mTORC2 in the temporal regulation of T_FH_ differentiation and effector function upon viral infection. mTORC1 responds to diverse environmental cues, including amino acids, stress, oxygen, energy, and growth factors (25), while mTORC2 is insensitive to nutrients but activated by growth factors (60); however, knowledge is scarce regarding mTORC2 stimuli compared with mTORC1. In our study, we demonstrated that the combination of ICOS and CD3 acted as upstream activators of mTORC2 signaling in T_FH_ cells. Moreover, we found that T_FH_ cells exhibited higher mTORC2 activity than T_H_1 cells upon stimulation, suggesting that T_FH_ cells might be dependent on mTORC2 activity to a greater degree. Accordingly, the generation of T_FH_ cells, not T_H_1 cells, was specifically impaired after deletion of *Rictor*, implying that T_FH_ cells were more sensitive to reduced mTORC2 activity. Furthermore, we found that mTORC2 was required for full differentiation of T_FH_ cells in the B cell-dependent phase, but not priming. In addition to T_FH_ generation, mTORC2 was also necessary for T_FH_ effector functions for helping B cells. Therefore, both lower total numbers and defective functions of T_FH_ cells synergistically resulted in aberrant humoral immunity, characterized by decreased GC B cells, a smaller GC size, poor production of virus-specific IgG, and diminished ASCs in BM. These data collectively indicated a crucial role of mTORC2 signaling in the full commitment of T_FH_ cells and humoral immunity.

Two groups recently reported that mTORC2 signaling regulates T_FH_ differentiation and GC responses in the mesenteric lymph nodes and PPs under the steady state as well as upon protein immunization *via* distinct mechanisms (41, 42). Yang and colleagues found that mTORC2 promoted T_FH_ cell survival but did not affect proliferation through the phosphorylation of AKT (42). In addition, they showed that the mTORC2–AKT–TCF-1 axis was important for T_FH_ differentiation (42). Another group showed that ICOS-mTORC2-Foxo1 signaling axis was required for T_FH_ differentiation by promoting glucose metabolism and the T_FH_ transcriptional program (41). Moreover, they observed a reduction of T_FH_ and GC B cells after abrogation of mTORC2 signaling in the LCMV acute infection model, which was repeatable in our study (41). However, these studies did not dissect the different roles of mTORC2 in early and late stages during T_FH_ differentiation, respectively, nor assess the influence of mTORC2 on T_FH_ effector functions. Here, we provided unambiguous evidence showing that mTORC2 signaling was required for the late, but early priming, stage of T_FH_ differentiation; in addition, mTORC2 was important for supporting T_FH_ effector functions.

The complete differentiation and maintenance of T_FH_ cells depends on the necessary signals, including CD80, CD86, ICOSL, and CD40, provided by cognate B cells (61–64). To receive signals from B cells, it is critical for T_FH_ cell to migrate to B cell follicles and colocalize with B cells after priming, and subsequently to form T cell–B cell junctions. Although we found that T cell–B cell adhesion was not affected by mTORC2, the T_FH_ migratory capacity in responses to CXCL13 was impaired in the absence of mTORC2, which resulted from downregulated CXCR5 expression. Furthermore, it has been reported that mTORC2 regulates cytoskeletal remodeling and cell migration by phosphorylating a series of proteins of PKC family, including PKC-α, PKC-δ, PKC-ζ, PKC-γ, and PKC-ε (65–69). Therefore, in addition to downregulated CXCR5 expression, mTORC2 may play a potential role in modulating T_FH_ cell migration *via* the mTORC2–PKC axis; however, the underlying mechanisms remain unclear and merit further exploration. Taken together, a compartmental segregation between T_FH_ and B cells formed after abrogation of mTORC2 signaling, preventing T_FH_ full differentiation in the B cell-dependent phase: fewer T_FH_ cells were maintained, underwent further development into GC T_FH_ cells, and accomplished functional maturation. Accordingly, we found that mTORC2-deficient T_FH_ cells failed to provide adequate help to B cells, even under *in vitro* culture conditions, which eliminated the interferences from reduced colocalization of T_FH_ and B cell. This indicated that mTORC2 signaling was critical for T_FH_ effector functions assisting B cells, at least in part because mTORC2 promoted the expression of T_FH_ function-related genes, such as *Il4, Il21*, and *Cd40lg*.

Our study results led to the conclusion that mTORC2 was involved in the regulation of T_FH_ cell late differentiation and effector functions. Moreover, it is well accepted that exaggerated T_FH_ responses and functions provide a great contribution to the pathogenesis of autoimmune diseases characterized by spontaneous GC formation and autoantibody production, such as systemic lupus erythematosus and rheumatoid arthritis (70–72). Therefore, suppression of T_FH_ responses by targeting the mTORC2 signaling pathway might serve as a potential therapeutic strategy for autoimmune diseases.

## Ethics Statement

All mouse experiments were performed in accordance with the guidelines of the Institutional Animal Care and Use Committees of the Third Military Medical University.

## Author Contributions

YH, ZY, YZW, and LY designed and oversaw experiments. YH, YFW, XL, XY, PW, QT, XC, ZL, JW, ZX, XZ, and YZ performed experiments. YH and QB analyzed experiments. YH and LY wrote the paper.

## Conflict of Interest Statement

The authors declare that the research was conducted in the absence of any commercial or financial relationships that could be construed as a potential conflict of interest.

## References

[1] Schaerli P Willimann K Lang AB Lipp M Loetscher P Moser B. CXC chemokine receptor 5 expression defines follicular homing T cells with B cell helper function. J Exp Med (2000) 192(11):1553–62. 10.1084/jem.192.11.1553 11104798 PMC2193097

[2] Kim CH Rott LS Clarklewis I Campbell DJ Wu L Butcher EC. Subspecialization of CXCR5+ T cells: B helper activity is focused in a germinal center-localized subset of CXCR5+ T cells. J Exp Med (2001) 193(12):1373–82. 10.1084/jem.193.12.1373 11413192 PMC2193300

[3] Breitfeld D Ohl L Kremmer E Ellwart JW Sallusto F Lipp M Follicular B helper T cells express Cxc chemokine receptor 5, localize to B cell follicles, and support immunoglobulin production. J Exp Med (2000) 192(11):1545–52. 10.1084/jem.192.11.1545 11104797 PMC2193094

[4] Cyster JG Ansel KM Reif K Ekland EH Hyman PL Tang HL Follicular stromal cells and lymphocyte homing to follicles. Immunol Rev (2000) 176:181–93. 10.1034/j.1600-065X.2000.00618.x 11043777

[5] Haynes NM Allen CDC Lesley R Ansel KM Killeen N Cyster JG. Role of CXCR5 and CCR7 in follicular Th cell positioning and appearance of a programmed cell death gene-1 high germinal center-associated subpopulation. J Immunol (2007) 179(8):5099–108. 10.4049/jimmunol.179.8.5099 17911595

[6] Ansel KM Mcheyzerwilliams LJ Ngo VN Mcheyzerwilliams MG Cyster JG In vivo-activated Cd4 T cells upregulate Cxc chemokine receptor 5 and reprogram their response to lymphoid chemokines. J Exp Med (1999) 190(8):1123–34. 10.1084/jem.190.8.1123 10523610 PMC2195660

[7] Crotty S. Follicular helper CD4 T cells (TFH). Annu Rev Immunol (2011) 29(1):621–63. 10.1146/annurev-immunol-031210-101400 21314428

[8] Victora GD Nussenzweig MC. Germinal centers. Annu Rev Immunol (2012) 30:429–57. 10.1146/annurev-immunol-020711-075032 22224772

[9] Goenka R Barnett LG Silver JS Neill PJO Hunter CA Cancro MP Cutting edge: dendritic cell-restricted antigen presentation initiates the follicular helper T cell program but cannot complete ultimate effector differentiation. J Immunol (2011) 187(3):1091–5. 10.4049/jimmunol.1100853 21715693 PMC3171798

[10] Johnston RJ Poholek AC DiToro D Yusuf I Eto D Barnett B Bcl6 and Blimp-1 are reciprocal and antagonistic regulators of T follicular helper cell differentiation. Science (2009) 325(5943):1006–10. 10.1126/science.1175870 19608860 PMC2766560

[11] Nurieva RI Chung Y Martinez GJ Yang XO Tanaka S Matskevitch TD Bcl6 mediates the development of T follicular helper cells. Science (2009) 325(5943):1001–5. 10.1126/science.1176676 19628815 PMC2857334

[12] Yu D Rao S Tsai LM Lee SK He Y Sutcliffe EL The transcriptional repressor Bcl-6 directs T follicular helper cell lineage commitment. Immunity (2009) 31(3):457–68. 10.1016/j.immuni.2009.07.002 19631565

[13] Choi YS Eto D Yang JA Lao C Crotty S Cutting edge: STAT1 is required for IL-6-mediated Bcl6 induction for early follicular helper cell differentiation. J Immunol (2013) 190(7):3049–53. 10.4049/jimmunol.1203032 23447690 PMC3626564

[14] Xu L Cao Y Xie Z Huang Q Bai Q Yang X The transcription factor TCF-1 initiates the differentiation of TFH cells during acute viral infection. Nat Immunol (2015) 16(9):991 10.1038/ni.3229 26214740

[15] Wu T Shin HM Moseman EA Ji Y Huang B Harly C TCF1 is required for the T follicular helper cell response to viral infection. Cell Rep (2015) 12(12):2099–110. 10.1016/j.celrep.2015.08.049 26365183 PMC4591235

[16] Choi YS Gullicksrud JA Xing S Zeng Z Shan Q Li F LEF-1 and TCF-1 orchestrate TFH differentiation by regulating differentiation circuits upstream of the transcriptional repressor Bcl6. Nat Immunol (2015) 16(9):980–90. 10.1038/ni.3226 26214741 PMC4545301

[17] Liu X Chen X Zhong B Wang A Wang X Chu F Transcription factor achaete-scute homologue 2 initiates follicular T-helper-cell development. Nature (2014) 507(7493):513–8. 10.1038/nature12910 24463518 PMC4012617

[18] Xu H Li X Liu D Li J Zhang X Chen X Follicular T-helper cell recruitment governed by bystander B cells and ICOS-driven motility. Nature (2013) 496(7446):523–7. 10.1038/nature12058 23619696

[19] Garside P Ingulli E Merica R Johnson JG Noelle RJ Jenkins MK. Visualization of specific B and T lymphocyte interactions in the lymph node. Science (1998) 281(5373):96–9. 10.1126/science.281.5373.96 9651253

[20] Okada T Miller MJ Parker I Krummel MF Neighbors M Hartley SB Antigen-engaged B cells undergo chemotaxis toward the T zone and form motile conjugates with helper T cells. PLoS Biol (2005) 3(6):e150. 10.1371/journal.pbio.0030150 15857154 PMC1088276

[21] Allen CDC Okada T Cyster JG. Germinal-center organization and cellular dynamics. Immunity (2007) 27(2):190–202. 10.1016/j.immuni.2007.07.009 17723214 PMC2242846

[22] Barnett LG Simkins HMA Barnett BE Korn LL Johnson AL Wherry EJ B cell antigen presentation in the initiation of follicular helper T cell and germinal center differentiation. J Immunol (2014) 192(8):3607–17. 10.4049/jimmunol.1301284 24646739 PMC4380085

[23] Choi YS Kageyama R Eto D Escobar TC Johnston RJ Monticelli L ICOS receptor instructs T follicular helper cell versus effector cell differentiation via induction of the transcriptional repressor Bcl6. Immunity (2011) 34(6):932–46. 10.1016/j.immuni.2011.03.023 21636296 PMC3124577

[24] Zoncu R Efeyan A Sabatini DM. mTOR: from growth signal integration to cancer, diabetes and ageing. Nat Rev Mol Cell Biol (2011) 12(1):21–35. 10.1038/nrm3025 21157483 PMC3390257

[25] Laplante M Sabatini DM. mTOR signaling in growth control and disease. Cell (2012) 149(2):274–93. 10.1016/j.cell.2012.03.017 22500797 PMC3331679

[26] Laplante M Sabatini DM mTOR signaling at a glance. J Cell Sci (2009) 122(20):3589–94. 10.1242/jcs.051011 19812304 PMC2758797

[27] Ma XM Blenis J. Molecular mechanisms of mTOR-mediated translational control. Nat Rev Mol Cell Biol (2009) 10(5):307–18. 10.1038/nrm2672 19339977

[28] Hay N Sonenberg N. Upstream and downstream of mTOR. Genes Dev (2004) 18(16):1926–45. 10.1101/gad.1212704 15314020

[29] Duvel K Yecies JL Menon S Raman P Lipovsky AI Souza AL Activation of a metabolic gene regulatory network downstream of mTOR complex 1. Mol Cell (2010) 39(2):171–83. 10.1016/j.molcel.2010.06.022 20670887 PMC2946786

[30] Oh WJ Jacinto E. mTOR complex 2 signaling and functions. Cell Cycle (2011) 10(14):2305–16. 10.4161/cc.10.14.16586 21670596 PMC3322468

[31] Chi H. Regulation and function of mTOR signalling in T cell fate decisions. Nat Rev Immunol (2012) 12(5):325–38. 10.1038/nri3198 22517423 PMC3417069

[32] Delgoffe GM Pollizzi KN Waickman AT Heikamp EB Meyers DJ Horton MR The kinase mTOR regulates the differentiation of helper T cells through the selective activation of signaling by mTORC1 and mTORC2. Nat Immunol (2011) 12(4):295–303. 10.1038/ni.2005 21358638 PMC3077821

[33] Battaglia M Stabilini A Roncarolo M. Rapamycin selectively expands CD4+CD25+FoxP3+ regulatory T cells. Blood (2005) 105(12):4743–8. 10.1182/blood-2004-10-3932 15746082

[34] Delgoffe GM Kole TP Zheng Y Zarek PE Matthews KL Xiao B The mTOR kinase differentially regulates effector and regulatory T cell lineage commitment. Immunity (2009) 30(6):832–44. 10.1016/j.immuni.2009.04.014 19538929 PMC2768135

[35] Haxhinasto S Mathis D Benoist C The AKT–mTOR axis regulates de novo differentiation of CD4+Foxp3+ cells. J Exp Med (2008) 205(3):565–74. 10.1084/jem.20071477 18283119 PMC2275380

[36] Liu G Burns S Huang G Boyd KL Proia RL Flavell RA The receptor S1P1 overrides regulatory T cell-mediated immune suppression through Akt-mTOR. Nat Immunol (2009) 10(7):769–77. 10.1038/ni.1743 19483717 PMC2732340

[37] Sauer S Bruno L Hertweck A Finlay DK Leleu M Spivakov M T cell receptor signaling controls Foxp3 expression via PI3K, Akt, and mTOR. Proc Natl Acad Sci U S A (2008) 105(22):7797–802. 10.1073/pnas.0800928105 18509048 PMC2409380

[38] Ray JP Staron M Shyer JA Ho P Marshall HD Gray SM The interleukin-2-mTORc1 kinase axis defines the signaling, differentiation, and metabolism of T helper 1 and follicular B helper T cells. Immunity (2015) 43(4):690–702. 10.1016/j.immuni.2015.08.017 26410627 PMC4618086

[39] Xu L Huang Q Wang H Hao Y Bai Q Hu J The kinase mTORC1 promotes the generation and suppressive function of follicular regulatory T cells. Immunity (2017) 47(3):538–51. 10.1016/j.immuni.2017.08.011 28930662

[40] Lee K Gudapati P Dragovic S Spencer CT Joyce S Killeen N Mammalian target of rapamycin protein complex 2 regulates differentiation of Th1 and Th2 cell subsets via distinct signaling pathways. Immunity (2010) 32(6):743–53. 10.1016/j.immuni.2010.06.002 20620941 PMC2911434

[41] Zeng H Cohen S Guy C Shrestha S Neale G Brown SA mTORC1 and mTORC2 kinase signaling and glucose metabolism drive follicular helper T cell differentiation. Immunity (2016) 45(3):540–54. 10.1016/j.immuni.2016.08.017 27637146 PMC5050556

[42] Yang J Lin X Pan Y Wang J Chen P Huang H Critical roles of mTOR Complex 1 and 2 for T follicular helper cell differentiation and germinal center responses. Elife (2016) 5:e17936. 10.7554/eLife.17936 27690224 PMC5063587

[43] Hale JS Youngblood B Latner DR Mohammed AUR Ye L Akondy R Distinct memory CD4+ T cells with commitment to T follicular helper- and T helper 1-cell lineages are generated after acute viral infection. Immunity (2013) 38(4):805–17. 10.1016/j.immuni.2013.02.020 23583644 PMC3741679

[44] Shen H Slifka MK Matloubian M Jensen ER Ahmed R Miller JF. Recombinant *Listeria monocytogenes* as a live vaccine vehicle for the induction of protective anti-viral cell-mediated immunity. Proc Natl Acad Sci U S A (1995) 92(9):3987–91. 10.1073/pnas.92.9.3987 7732018 PMC42087

[45] Rasheed MAU Latner DR Aubert RD Gourley T Spolski R Davis CW Interleukin-21 is a critical cytokine for the generation of virus-specific long-lived plasma cells. J Virol (2013) 87(13):7737–46. 10.1128/JVI.00063-13 23637417 PMC3700268

[46] Hao Y Li Z Wang Y Liu X Ye L Analyzing mouse B cell responses specific to LCMV infection. In: Liu C, editor. B Cell Receptor Signaling: Methods and Protocols, Methods in Molecular Biology. Vol 1707 New York: Springer (2018). p. 15–38. 10.1007/978-1-4939-7474-0_2 29388097

[47] Subramanian A Tamayo P Mootha VK Mukherjee S Ebert BL Gillette MA Gene set enrichment analysis: a knowledge-based approach for interpreting genome-wide expression profiles. Proc Natl Acad Sci U S A (2005) 102(43):15545–50. 10.1073/pnas.0506580102 16199517 PMC1239896

[48] Edgar R Domrachev M Lash AE. Gene expression omnibus: NCBI gene expression and hybridization array data repository. Nucleic Acids Res (2002) 30(1):207–10. 10.1093/nar/30.1.207 11752295 PMC99122

[49] Oxenius A Bachmann MF Zinkernagel RM Hengartner H Virus-specific major MHC class II-restricted TCR-transgenic mice: effects on humoral and cellular immune responses after viral infection. Eur J Immunol (1998) 28(1):390–400. 10.1002/(SICI)1521-4141(199801)28:01<390::AID-IMMU390>3.0.CO;2-O 9485218

[50] Fuller KA Kanagawa O Nahm MH. T cells within germinal centers are specific for the immunizing antigen. J Immunol (1993) 151(9):4505–12. 7691953

[51] Qi H Cannons JL Klauschen F Schwartzberg PL Germain RN. SAP-controlled T-B cell interactions underlie germinal centre formation. Nature (2008) 455(7214):764–9. 10.1038/nature07345 18843362 PMC2652134

[52] Han S Hathcock K Zheng B Kepler TB Hodes R Kelsoe G. Cellular interaction in germinal centers. Roles of CD40 ligand and B7-2 in established germinal centers. J Immunol (1995) 155(2):556–67. 7541819

[53] Kelsoe G, editor. The germinal center: a crucible for lymphocyte selection. Semin Immunol (1996) 8(3):179–84. 10.1006/smim.1996.0022 8738917

[54] Maclennan ICM Toellner K Cunningham AF Serre K Sze DMY Zuniga E Extrafollicular antibody responses. Immunol Rev (2003) 194(1):8–18. 10.1034/j.1600-065X.2003.00058.x 12846803

[55] Kerfoot SM Yaari G Patel JR Johnson KL Gonzalez D Kleinstein SH Germinal center B cell and T follicular helper cell development initiates in the interfollicular zone. Immunity (2011) 34(6):947–60. 10.1016/j.immuni.2011.03.024 21636295 PMC3280079

[56] Choi YS Yang JA Yusuf I Johnston RJ Greenbaum J Peters B Bcl6 expressing follicular helper CD4 T cells are fate committed early and have the capacity to form memory. J Immunol (2013) 190(8):4014–26. 10.4049/jimmunol.1202963 23487426 PMC3626566

[57] Linterman MA Beaton LL Yu D Ramiscal RR Srivastava M Hogan JJ IL-21 acts directly on B cells to regulate Bcl-6 expression and germinal center responses. J Exp Med (2010) 207(2):353–63. 10.1084/jem.20091738 20142429 PMC2822609

[58] Zotos D Coquet JM Zhang Y Light A Dcosta K Kallies A IL-21 regulates germinal center B cell differentiation and proliferation through a B cell-intrinsic mechanism. J Exp Med (2010) 207(2):365–78. 10.1084/jem.20091777 20142430 PMC2822601

[59] Eto D Lao C Ditoro D Barnett B Escobar TC Kageyama R IL-21 and IL-6 are critical for different aspects of B cell immunity and redundantly induce optimal follicular helper CD4 T cell (Tfh) differentiation. PLoS One (2011) 6(3):e17739. 10.1371/journal.pone.0017739 21423809 PMC3056724

[60] Zinzalla V Stracka D Oppliger W Hall MN. Activation of mTORC2 by association with the ribosome. Cell (2011) 144(5):757–68. 10.1016/j.cell.2011.02.014 21376236

[61] Nurieva R Chung Y Hwang D Yang XO Kang HS Ma L Generation of T follicular helper cells is mediated by interleukin-21 but independent of T helper 1, 2, or 17 cell lineages. Immunity (2008) 29(1):138–49. 10.1016/j.immuni.2008.05.009 18599325 PMC2556461

[62] Salekardakani S Choi YS Benhnia MR Flynn R Arens R Shoenberger S B cell-specific expression of B7-2 is required for follicular Th cell function in response to vaccinia virus. J Immunol (2011) 186(9):5294–303. 10.4049/jimmunol.1100406 21441451 PMC3089765

[63] Goodjacobson KL Song E Anderson SM Sharpe AH Shlomchik MJ. CD80 expression on B cells regulates murine T follicular helper development, germinal center B cell survival, and plasma cell generation. J Immunol (2012) 188(9):4217–25. 10.4049/jimmunol.1102885 22450810 PMC3331930

[64] Linterman MA Denton AE Divekar DP Zvetkova I Kane L Ferreira C CD28 expression is required after T cell priming for helper T cell responses and protective immunity to infection. Elife (2014) 3:e03180. 10.7554/eLife.03180 25347065 PMC4241536

[65] Sarbassov DD Ali SM Kim D Guertin DA Latek RR Erdjumentbromage H Rictor, a novel binding partner of mTOR, defines a rapamycin-insensitive and raptor-independent pathway that regulates the cytoskeleton. Curr Biol (2004) 14(14):1296–302. 10.1016/j.cub.2004.06.054 15268862

[66] Jacinto E Loewith R Schmidt A Lin S Ruegg MA Hall A Mammalian TOR complex 2 controls the actin cytoskeleton and is rapamycin insensitive. Nat Cell Biol (2004) 6(11):1122–8. 10.1038/ncb1183 15467718

[67] Gan X Wang J Wang C Sommer E Kozasa T Srinivasula SM PRR5L degradation promotes mTORC2-mediated PKC-δ phosphorylation and cell migration downstream of Gα12. Nat Cell Biol (2012) 14(7):686–96. 10.1038/ncb2507 22609986 PMC3389271

[68] Li X Gao T mTORC2 phosphorylates protein kinase Cζ to regulate its stability and activity. EMBO Rep (2014) 15(2):191–8. 10.1002/embr.201338119 24375676 PMC3989865

[69] Thomanetz V Angliker N Cloetta D Lustenberger RM Schweighauser M Oliveri F Ablation of the mTORC2 component Rictor in brain or Purkinje cells affects size and neuron morphology. J Cell Biol (2013) 201(2):293–308. 10.1083/jcb.201205030 23569215 PMC3628512

[70] Ueno H. T follicular helper cells in human autoimmunity. Curr Opin Immunol (2016) 43:24–31. 10.1016/j.coi.2016.08.003 27588918

[71] Jeon Y Choi YS. Follicular helper T (Tfh) cells in autoimmune diseases and allograft rejection. Immune Netw (2016) 16(4):219–32. 10.4110/in.2016.16.4.219 27574501 PMC5002448

[72] Crotty S. T follicular helper cell differentiation, function, and roles in disease. Immunity (2014) 41(4):529–42. 10.1016/j.immuni.2014.10.004 25367570 PMC4223692

