# Supplementary material for: The Kinase Complex mTOR Complex 2 Promotes the Follicular Migration and Functional Maturation of Differentiated Follicular Helper CD4+ T Cells During Viral Infection
Source: Front Immunol. 2018 May 23;9:1127. doi: 10.3389/fimmu.2018.01127 (PMC5974104; doi:10.3389/fimmu.2018.01127)
Supplement: Supplementary file 1 [file data_sheet_1.docx]

Supplementary Material

The kinase complex mTORC2 promotes the follicular migration and functional maturation of differentiated T_FH_ cells during viral infection

Yaxing Hao^*^, Yifei Wang, Xiaobing Liu, Xia Yang, Pengcheng Wang, Qin Tian, Qiang Bai, Xiangyu Chen, Zhirong Li, Jialin Wu, Zhunyi Xie, Xinyuan Zhou, Yuyang Zhou, Zhinan Yin, Yuzhang Wu, Lilin Ye

*** Correspondence:** Zhinan Yin, Yuzhang Wu, Lilin Ye: [zhinan.yin@yale.edu](mailto:zhinan.yin@yale.edu); wuyuzhang@tmmu.edu.cn; [yelilinlcmv@tmmu.edu.cn](mailto:yelilinlcmv@tmmu.edu.cn)

# Supplementary Figures and Tables

## Supplementary Figures


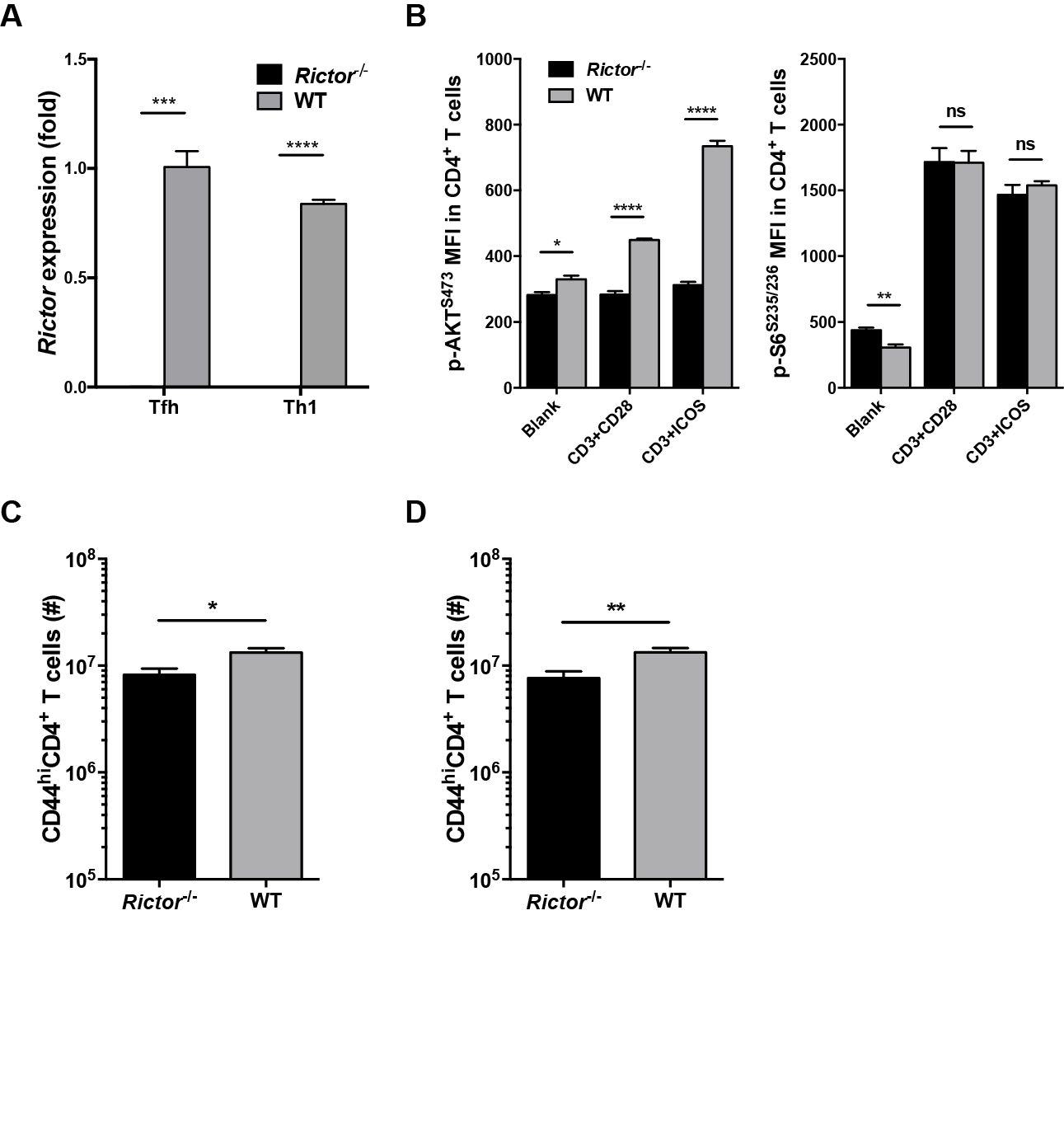


**Supplementary Figure 1.** Deletion efficiency and CD4^+^ T cell activation in *Rictor*^-/-^ versus WT mice. (**A**) RNA level of *Rictor* in T_FH_ and T_H_1 cells sorted from *Rictor*^-/-^ and WT mice at day 8 after LCMV infection (n=3 samples per group). (**B**) p-AKT^S473^ (**left**) and p-S6^S235/236^ (**right**) MFI in CD4^+^ T cells upon stimulation in *Rictor*^-/-^ versus WT mice at day 8 after LCMV infection (n=5 mice per group). (**C, D**) Quantification of the absolute number of CD44^hi^CD4^+^ T cells from *Rictor*^-/-^ and WT mice at day 8 after LCMV infection in gp66-tetramer specific (**C**) and bulk (**D**) T_FH_ staining panels, respectively (n=8 mice per group). ns, not significant, *p < 0.05, **p < 0.01, ***p < 0.001, ****p < 0.0001 (unpaired two-tailed t-test (**A-D**)). Data are representative of two (**A**) or three (**B**) or four (**C, D**) independent experiments. Error bars are SEM (**A-D**).


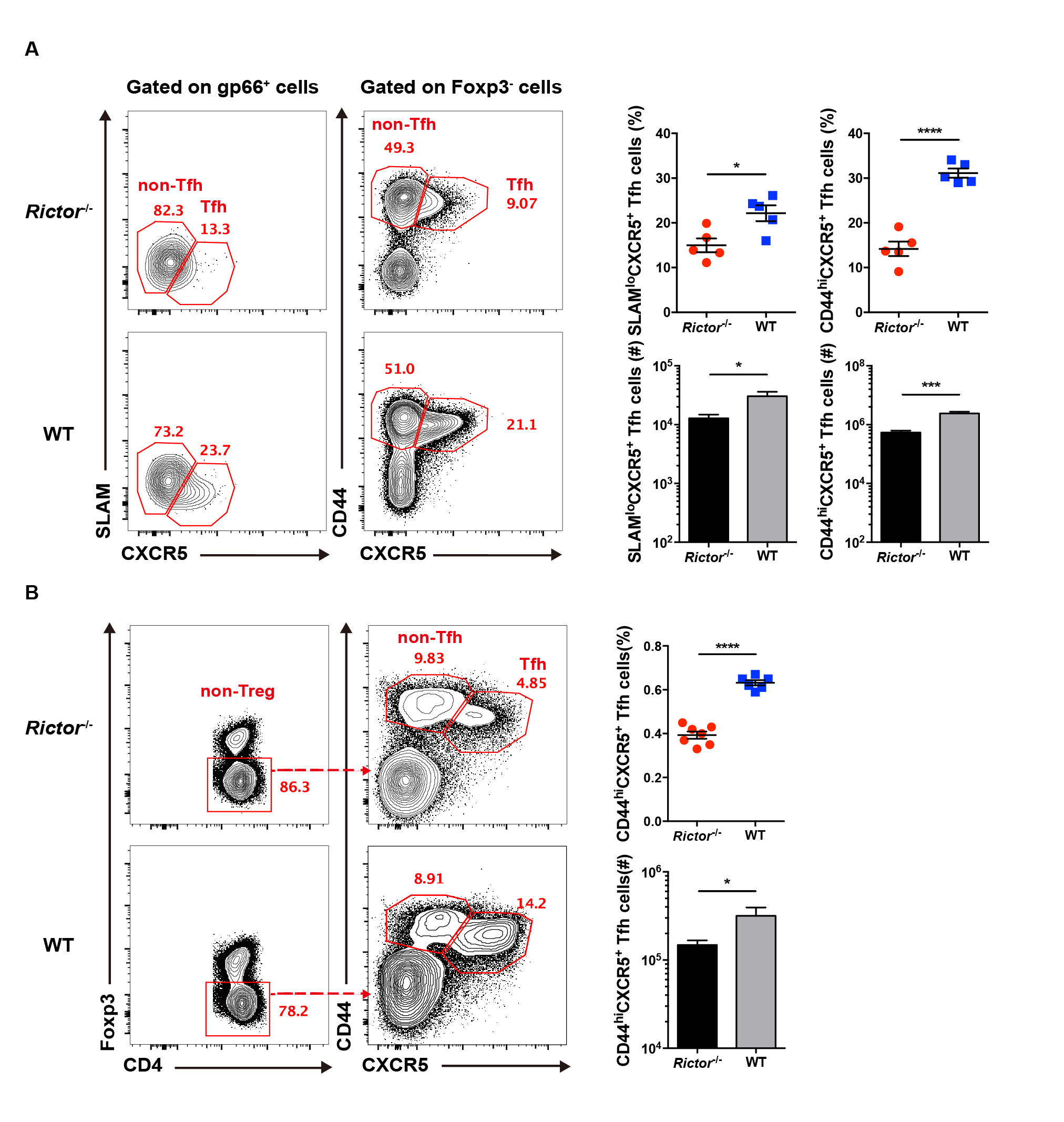


**Supplementary Figure 2.** mTORC2 is required for T_FH_ differentiation during listeria infection and protein immunization. (**A**) *Rictor*^-/-^ and WT mice were infected with listeria monocytogenes expressing LCMV gp61-80 (LM-gp61) and assessed at day 8 after infection. Flow cytometry plots of T_FH_ cells (SLAM^lo^CXCR5^+^) among gp66 tetramer-positive CD4^+^ T cells and bulk activated T_FH_ cells (CD44^hi^CXCR5^+^) among Foxp3^-^CD4^+^ T cells (**left**). Summary of the frequencies and cell numbers of tetramer-positive and bulk T_FH_ cells (**right**) (n=5 mice per group). (**B**) Flow cytometry of T_FH_ cells (CD44^hi^CXCR5^+^) among Foxp3^-^CD4^+^ T cells in *Rictor*^-/-^ and WT mice at day 8 after NP-OVA/CFA immunization (**left**). Quantification of the frequency and cell number of T_FH_ cells (**right**) (n=6-7 mice per group). *p < 0.05, ***p < 0.001, ****p < 0.0001 (unpaired two-tailed t-test (**A, B**)). Data are representative of two (**A, B**) independent experiments. Error bars are SEM (**A, B**).


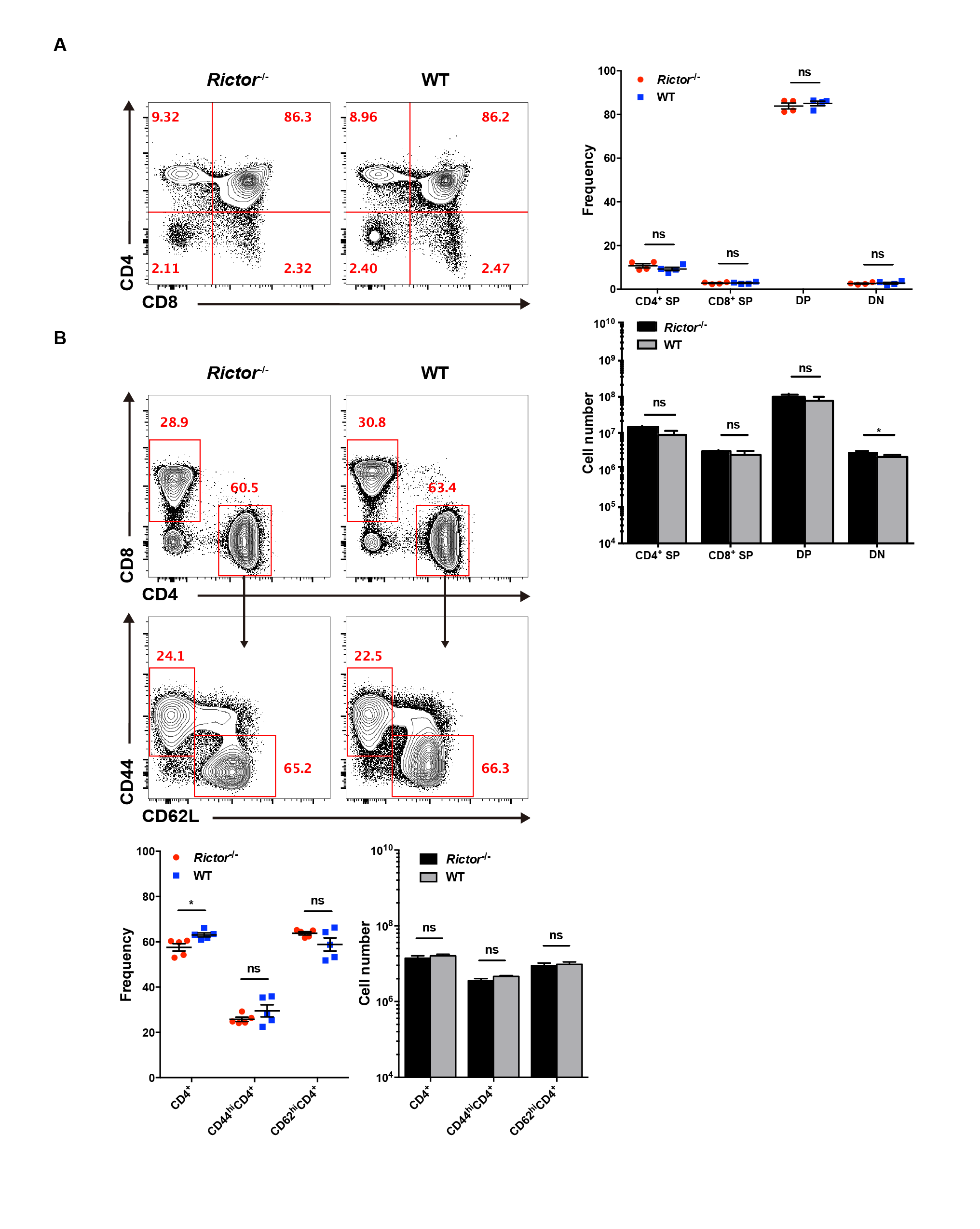


**Supplementary Figure 3.** mTORC2 is irresponsible for T cell development and immune homeostasis in the naive state. (**A**) Flow cytometry of CD4^+^ SP, CD8^+^ SP, DP, and DN T cells in thymus from naive *Rictor*^-/-^ and WT mice (**left**) and summary of the frequency and cell number (**right**) (SP: single positive, DP: double positive, DN: double negative) (n=4 mice per group). (**B**) Flow cytometry of total CD4^+^ T, CD44^hi^CD4^+^ and CD62^hi^CD4^+^ T cells in spleen from naive *Rictor*^-/-^ and WT mice (**top**) and summary of the frequency and cell number (**bottom**) (n=5 mice per group). ns, not significant, *p < 0.05 (unpaired two-tailed t-test (**A, B**)). Data are representative of two (**A, B**) independent experiments. Error bars are SEM (**A, B**).


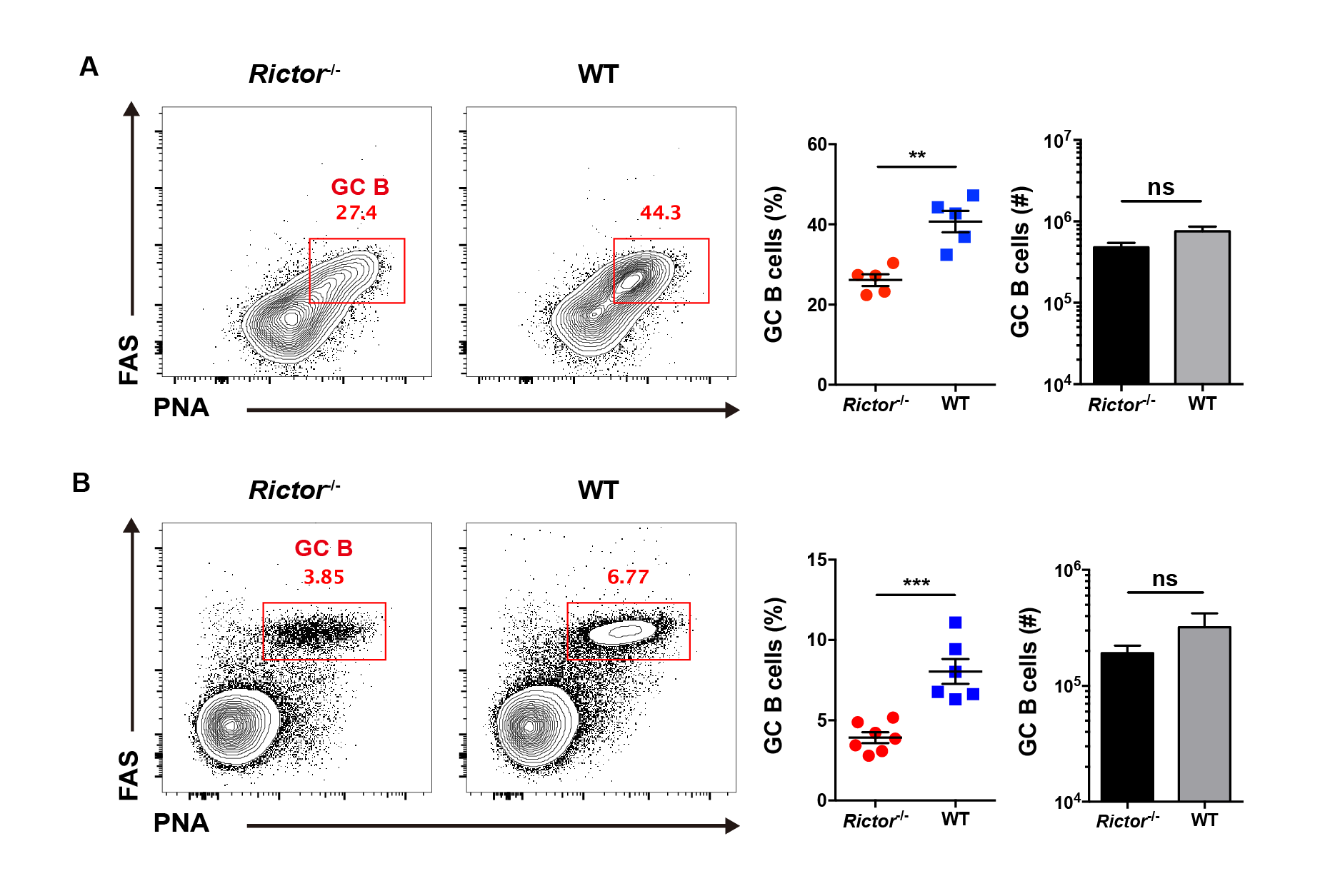


**Supplementary Figure 4.** Differentiation of GC B cell is constrained in the absence of mTORC2 signaling during listeria infection and protein immunization. (**A**) *Rictor*^-/-^ and WT mice were infected with LM-gp61 and assessed at day 8 after infection. Flow cytometry plots of GC B cells (PNA^hi^FAS^hi^) (**left**) and summary of the frequency and cell number of GC B cells (**right**) (n=5 mice per group). (**B**) Flow cytometry of GC B cells (PNA^hi^FAS^hi^) in *Rictor*^-/-^ and WT mice at day 8 after NP-OVA/CFA immunization (**left**). Quantification of the frequency and cell number of GC B cells (**right**) (n=6-7 mice per group). ns, not significant, **p < 0.01, ***p < 0.001 (unpaired two-tailed t-test (**A, B**)). Data are representative of two (**A, B**) independent experiments. Error bars are SEM (**A, B**).


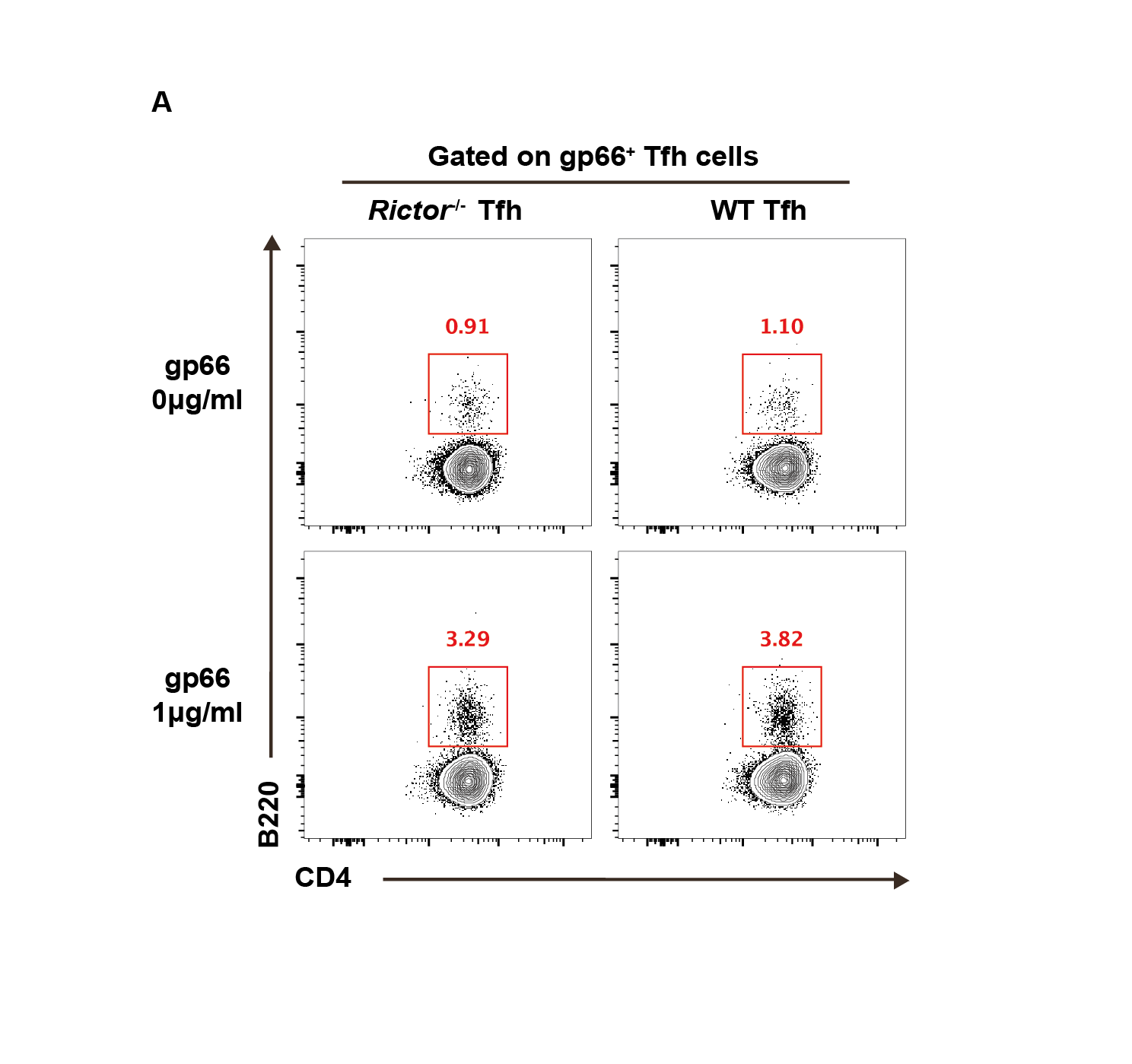


**Supplementary Figure 5.** mTORC2 is dispensable for T cell-B cell adhesion. (**A**) Flow cytometry of T cell-B cell conjugates. Gp66 tetramer-positive T_FH_ cells were incubated with LPS-activated B cells pulsed with cognate gp66-peptide or unpulsed. Data are representative of two independent experiments.

## Supplementary tables

**Supplementary table 1. Antibodies and reagents used in flow cytometry**

| **Antibody target/Reagent** | **Clone/Cat.#** | **Provider** |
| --- | --- | --- |
| CD4 | RM4-5 | Biolegend |
| CD8 | 53-6.7 | BD Biosciences |
| CD19 | 6D5 | Biolegend |
| CD25 | PC61.5 | Biolegend |
| CD44 | IM7 | eBioscience |
| CD45.1 | A20 | Biolegend |
| CD45.2 | 104 | Biolegend |
| CD62L | MEL-14 | eBioscience |
| Tim3 | 215008 | R&D Systems |
| SLAM | TC15-12F12.2 | Biolegend |
| ICOS | C398.4A | Biolegend |
| PD-1 | RMP1-30 | eBioscience |
| GITR | DTA-1 | eBioscience |
| B220 | RA3-6B2 | eBioscience |
| PNA | FL-1071 | Vector Labs |
| FAS | JO2 | BD Biosciences |
| CD138 | 281-2 | BD Biosciences |
| IgD | 11-26c.2a | BD Biosciences |
| GL-7 | GL7 | BD Biosciences |
| I-A/I-E | M5/114.15.2 | eBioscience |
| CXCR5 | 2G8 | BD Biosciences |
| Biotin Goat anti-Rat IgG | 112-065-143 | Jackson Immunoresearch |
| Streptavidin | 25-4317-82 | eBioscience |
| Live/Dead Kit | L10119 | Life Technologies |
| Fc-blocker | 2.4G2 | BD Biosciences |
| Foxp3 | FJK-16s | eBioscience |
| Bcl-6 | K112-91 | BD Biosciences |
| c-Maf | symoF1 | eBioscience |
| TCF-1 | C46C7 | Cell Signalling Technology |
| IgG1 | A85-1 | BD Biosciences |
| IgG2a | eBM2a | eBioscience |
| p-AKT(S473) | D9E | Cell Signalling Technology |
| p-S6(S235/236) | D57.2.2E | Cell Signalling Technology |
| anti-rabbit IgG A488 | A21206 | Invitrogen |
| anti-rabbit IgG A647 | 4414S | Cell Signalling Technology |

**Supplementary table 2. Primers used in quantitative PCR**

| **Gene symbol** | **Forward primer** | **Reverse primer** |
| --- | --- | --- |
| ***Ascl2*** | tactcgtcggaggaaagcag | acccagggatgcagcttag |
| ***Bcl6*** | agacgcacagtgacaaacca | agtgtgggtcttcaggttgg |
| ***Cd40lg*** | ctcaaattgcagcacacgtt | cgtcagctgtttcccatttt |
| ***Cxcr5*** | catgggctccatcacataca | ggcatgaataccgccttaaa |
| ***Hprt*** | gcgtcgtgattagcgatgatg | ctcgagcaagtctttcagtcc |
| ***Il4*** | ggcattttgaacgaggtcac | aaatatgcgaagcaccttgg |
| ***Il6st*** | catgctttcaggctttcctc | tcccactggcacagcatatt |
| ***Il6ra*** | gcaagaatcctcgtccatgt | gtggaggagaggtcgtcttg |
| ***Il21r*** | ccccgtcatctttcagaccc | ggcactggtgcccatatctt |
| ***Il21*** | cgcctcctgattagacttcg | aaaacaggcaaaagctgcat |
| ***Maf*** | aaggaggaggtgatccgact | tctcctgcttgaggtggtct |
| ***Pdcd1*** | gcaggtaccctggtcattca | caggctgggtagaaggtgag |
| ***Rictor*** | tatgtaagcctggaaattctg | gcagggcttctatgaactca |
| ***Tcf7*** | caatctgctcatgccctacc | cttgcttctggctgatgtcc |

**Supplementary table 3. Gene expression profiles of *Rictor*^-/-^ and WT T_FH_ cells**

| **Gene Symbol** | **Gene Title** | **Fold change (*Rictor*^-/-^ / WT)** |
| --- | --- | --- |
| ***Gdpd3*** | glycerophosphodiester phosphodiesterase domain containing 3 | 25.0341 |
| ***Gzma*** | granzyme A | 5.1632 |
| ***Ccl3*** | chemokine (C-C motif) ligand 3 | 4.1053 |
| ***Fam135a*** | "family with sequence similarity 135, member A" | 3.9861 |
| ***Hba-a1 /// Hba-a2*** | "hemoglobin alpha, adult chain 1 /// hemoglobin alpha, adult chain 2" | 3.9815 |
| ***Aicda*** | activation-induced cytidine deaminase | 3.7575 |
| ***Pik3ap1*** | phosphoinositide-3-kinase adaptor protein 1 | 3.6608 |
| ***Ifitm1*** | interferon induced transmembrane protein 1 | 3.4708 |
| ***Rpl39l*** | ribosomal protein L39-like | 3.4475 |
| ***Plac8*** | placenta-specific 8 | 3.4203 |
| ***Irak3*** | interleukin-1 receptor-associated kinase 3 | 3.3967 |
| ***Igk-V28*** | immunoglobulin kappa chain variable 28 (V28) | 3.2613 |
| ***Fcgr3*** | "Fc receptor, IgG, low affinity III" | 3.1657 |
| ***Tppp3*** | tubulin polymerization-promoting protein family member 3 | 3.1299 |
| ***Iglv1*** | immunoglobulin lambda variable 1 | 3.0878 |
| ***Igj*** | immunoglobulin joining chain | 3.0561 |
| ***Fgl2*** | fibrinogen-like protein 2 | 3.0518 |
| ***Gp49a /// Lilrb4*** | "glycoprotein 49 A /// leukocyte immunoglobulin-like receptor, subfamily B, member 4" | 3.0382 |
| ***AA467197*** | expressed sequence AA467197 | 2.9992 |
| ***Klrk1*** | "killer cell lectin-like receptor subfamily K, member 1" | 2.9517 |
| ***Wdr95*** | WD40 repeat domain 95 | 2.8930 |
| ***Itgax*** | integrin alpha X | 2.8758 |
| ***Ccr5*** | chemokine (C-C motif) receptor 5 | 2.8664 |
| ***abParts /// Igkv6-13 /// Igkv6-14*** | "Parts of antibodies, mostly variable regions. /// immunoglobulin kappa variable 6-13 /// immunoglobulin kappa variable 6-14" | 2.8541 |
| ***abParts /// Igk /// Igkv3-12*** | "Parts of antibodies, mostly variable regions. /// immunoglobulin kappa chain complex /// immunoglobulin kappa variable 3-12" | 2.8538 |
| ***Rps4l*** | ribosomal protein S4-like | 2.8313 |
| ***Skiv2l2*** | superkiller viralicidic activity 2-like 2 (S. cerevisiae) | 2.8122 |
| ***Ermn*** | "ermin, ERM-like protein" | 2.8039 |
| ***2310031A07Rik*** | RIKEN cDNA 2310031A07 gene | 2.7900 |
| ***Tmprss13*** | "transmembrane protease, serine 13" | 2.7548 |
| ***Tktl1*** | transketolase-like 1 | 2.7476 |
| ***Klrg1*** | "killer cell lectin-like receptor subfamily G, member 1" | 2.7215 |
| ***Iglc1 /// Iglv1*** | immunoglobulin lambda constant 1 /// immunoglobulin lambda variable 1 | 2.7179 |
| ***Gzmk*** | granzyme K | 2.6794 |
| ***Havcr2*** | hepatitis A virus cellular receptor 2 | 2.6447 |
| ***Hbb-b1 /// Hbb-b2 /// Hbb-bs /// Hbb-bt*** | "hemoglobin, beta adult major chain /// hemoglobin, beta adult minor chain /// hemoglobin, beta adult s chain /// hemoglobin, beta adult t chain" | 2.6393 |
| ***2010309G21Rik*** | RIKEN cDNA 2010309G21 gene | 2.6279 |
| ***Ccl4*** | chemokine (C-C motif) ligand 4 | 2.6169 |
| ***Cdkn3*** | cyclin-dependent kinase inhibitor 3 | 2.6105 |
| ***Chil3*** | chitinase-like 3 | 2.5172 |
| ***Igkv8-30*** | immunoglobulin kappa chain variable 8-30 | 2.5144 |
| ***6230424C14Rik*** | RIKEN cDNA 6230424C14 gene | 2.5136 |
| ***Socs2*** | suppressor of cytokine signaling 2 | 2.4984 |
| ***Gpr160*** | G protein-coupled receptor 160 | 2.4710 |
| ***Ctla2b*** | cytotoxic T lymphocyte-associated protein 2 beta | 2.4387 |
| ***Apobec2*** | "apolipoprotein B mRNA editing enzyme, catalytic polypeptide 2" | 2.4372 |
| ***Prss12*** | "protease, serine 12 neurotrypsin (motopsin)" | 2.4282 |
| ***Akr1c12 /// Akr1c13*** | "aldo-keto reductase family 1, member C12 /// aldo-keto reductase family 1, member C13" | 2.4076 |
| ***Igk-V28 /// Igkc /// Igkj1 /// Igkv4-53 /// Igkv6-23 /// Igkv8-30*** | immunoglobulin kappa chain variable 28 (V28) /// immunoglobulin kappa constant /// immunoglobulin kappa joining 1 /// immunoglobulin kappa variable 4-53 /// immunoglobulin kappa variable 6-23 /// immunoglobulin kappa chain variable 8-30 | 2.4028 |
| ***Kif20a*** | kinesin family member 20A | 2.3946 |
| ***As3mt*** | arsenic (+3 oxidation state) methyltransferase | 2.3759 |
| ***Klrd1*** | "killer cell lectin-like receptor, subfamily D, member 1" | 2.3484 |
| ***Igk*** | immunoglobulin kappa chain complex | 2.3426 |
| ***Igk-V1 /// Igkv9-120*** | immunoglobulin kappa chain variable 1 (V1) /// immunoglobulin kappa chain variable 9-120 | 2.3402 |
| ***Ccnb1 /// Gm5593*** | cyclin B1 /// predicted gene 5593 | 2.3155 |
| ***Mis18bp1*** | MIS18 binding protein 1 | 2.3096 |
| ***Dapl1*** | death associated protein-like 1 | 2.3045 |
| ***Klre1*** | killer cell lectin-like receptor family E member 1 | 2.2866 |
| ***Gstt1*** | "glutathione S-transferase, theta 1" | 2.2770 |
| ***9430064K01Rik*** | RIKEN cDNA 9430064K01 gene | 2.2649 |
| ***Ahsp*** | alpha hemoglobin stabilizing protein | 2.2617 |
| ***Il1r2*** | "interleukin 1 receptor, type II" | 2.2537 |
| ***Cd86*** | CD86 antigen | 2.2518 |
| ***Fcgr2b*** | "Fc receptor, IgG, low affinity IIb" | 2.2512 |
| ***Cenpp*** | centromere protein P | 2.2484 |
| ***Casc5*** | cancer susceptibility candidate 5 | 2.2411 |
| ***Rras2*** | related RAS viral (r-ras) oncogene homolog 2 | 2.2129 |
| ***Plek*** | pleckstrin | 2.2119 |
| ***S1pr5*** | sphingosine-1-phosphate receptor 5 | 2.2111 |
| ***Cenpf*** | centromere protein F | 2.2106 |
| ***Pcsk1*** | proprotein convertase subtilisin/kexin type 1 | 2.2040 |
| ***Lrrk1*** | leucine-rich repeat kinase 1 | 2.1947 |
| ***Ier3*** | immediate early response 3 | 2.1927 |
| ***Ighg3*** | Immunoglobulin heavy constant gamma 3 | 2.1917 |
| ***Itgam*** | integrin alpha M | 2.1886 |
| ***Ctla2a /// Ctla2b*** | cytotoxic T lymphocyte-associated protein 2 alpha /// cytotoxic T lymphocyte-associated protein 2 beta | 2.1830 |
| ***Sell*** | "selectin, lymphocyte" | 2.1829 |
| ***Spag5*** | sperm associated antigen 5 | 2.1788 |
| ***Tcrg /// Tcrg /// Tcrg-C2 /// Tcrg-C3 /// Tcrg-V4 /// Trgv2*** | "Mus musculus 2 days neonate thymus thymic cells cDNA, RIKEN full-length enriched library, clone:E430032M10 product:T-cell receptor gamma-1 chain C region homolog [Mus musculus], full insert sequence. /// Mus musculus cDNA clone IMAGE:1396673. /// T-cell receptor gamma, constant 2 /// SubName: Full=Protein Tcrg-C3; /// T cell receptor gamma, variable 4 /// T cell receptor gamma variable 2" | 2.1671 |
| ***Tmem159*** | transmembrane protein 159 | 2.1663 |
| ***Gas7*** | growth arrest specific 7 | 2.1635 |
| ***2810408I11Rik*** | RIKEN cDNA 2810408I11 gene | 2.1625 |
| ***Esco2*** | establishment of cohesion 1 homolog 2 (S. cerevisiae) | 2.1547 |
| ***Lrr1*** | leucine rich repeat protein 1 | 2.1547 |
| ***Mzb1*** | marginal zone B and B1 cell-specific protein 1 | 2.1443 |
| ***Cep55*** | centrosomal protein 55 | 2.1367 |
| ***H2-Aa*** | "histocompatibility 2, class II antigen A, alpha" | 2.1284 |
| ***Ank3*** | "ankyrin 3, epithelial" | 2.1143 |
| ***Rad51ap1*** | RAD51 associated protein 1 | 2.1113 |
| ***Dyx1c1*** | dyslexia susceptibility 1 candidate 1 homolog (human) | 2.1035 |
| ***Igh /// Ighg /// Ighg2c /// Ighm*** | RecName: Full=Ig gamma-2A chain C region secreted form; AltName: Full=B allele; /// RecName: Full=Ig gamma-2A chain C region secreted form; AltName: Full=B allele; /// immunoglobulin heavy constant gamma 2C /// immunoglobulin heavy constant mu | 2.0951 |
| ***Igk-V28 /// Igkv6-14 /// Igkv6-23 /// Igkv8-19*** | immunoglobulin kappa chain variable 28 (V28) /// immunoglobulin kappa variable 6-14 /// immunoglobulin kappa variable 6-23 /// immunoglobulin kappa variable 8-19 | 2.0935 |
| ***Spc25*** | "SPC25, NDC80 kinetochore complex component, homolog (S. cerevisiae)" | 2.0934 |
| ***Cenpw*** | centromere protein W | 2.0864 |
| ***Cenpi*** | centromere protein I | 2.0798 |
| ***Nusap1*** | nucleolar and spindle associated protein 1 | 2.0794 |
| ***Klra3 /// Klra9 /// LOC100862437*** | "killer cell lectin-like receptor, subfamily A, member 3 /// killer cell lectin-like receptor subfamily A, member 9 /// killer cell lectin-like receptor 3-like" | 2.0764 |
| ***Myl4*** | "myosin, light polypeptide 4" | 2.0740 |
| ***Mettl20*** | methyltransferase like 20 | 2.0701 |
| ***Slc2a3*** | "solute carrier family 2 (facilitated glucose transporter), member 3" | 2.0667 |
| ***Wfikkn2*** | "WAP, follistatin/kazal, immunoglobulin, kunitz and netrin domain containing 2" | 2.0632 |
| ***4930486L24Rik*** | RIKEN cDNA 4930486L24 gene | 2.0585 |
| ***Dnajc15*** | "DnaJ (Hsp40) homolog, subfamily C, member 15" | 2.0462 |
| ***Cdk1*** | cyclin-dependent kinase 1 | 2.0392 |
| ***Cx3cr1*** | chemokine (C-X3-C motif) receptor 1 | 2.0369 |
| ***Entpd1*** | ectonucleoside triphosphate diphosphohydrolase 1 | 2.0286 |
| ***Shcbp1*** | Shc SH2-domain binding protein 1 | 2.0280 |
| ***Sgol1*** | shugoshin-like 1 (S. pombe) | 2.0279 |
| ***Pvt1*** | plasmacytoma variant translocation 1 | 2.0252 |
| ***Ighg*** | Immunoglobulin heavy chain (gamma polypeptide) | 2.0117 |
| ***Cd55*** | CD55 antigen | 2.0085 |
| ***Slc25a33*** | "solute carrier family 25, member 33" | 2.0083 |
| ***Cenpk*** | centromere protein K | 2.0080 |
| ***Pde2a*** | "phosphodiesterase 2A, cGMP-stimulated" | 2.0038 |
| ***Ctla2a*** | cytotoxic T lymphocyte-associated protein 2 alpha | 2.0005 |
| ***Ly6k*** | "lymphocyte antigen 6 complex, locus K" | 1.9946 |
| ***Susd1*** | sushi domain containing 1 | 1.9905 |
| ***Il2*** | interleukin 2 | 1.9794 |
| ***Serp2*** | stress-associated endoplasmic reticulum protein family member 2 | 1.9754 |
| ***Tspan2*** | tetraspanin 2 | 1.9736 |
| ***Ncapg*** | "non-SMC condensin I complex, subunit G" | 1.9727 |
| ***Car2*** | carbonic anhydrase 2 | 1.9663 |
| ***Acyp2*** | "acylphosphatase 2, muscle type" | 1.9516 |
| ***Knstrn*** | kinetochore-localized astrin/SPAG5 binding | 1.9436 |
| ***Epsti1*** | epithelial stromal interaction 1 (breast) | 1.9364 |
| ***Serpinb1b*** | "serine (or cysteine) peptidase inhibitor, clade B, member 1b" | 1.9341 |
| ***Eri2*** | exoribonuclease 2 | 1.9329 |
| ***1700019D03Rik*** | RIKEN cDNA 1700019D03 gene | 1.9305 |
| ***Cgrrf1*** | cell growth regulator with ring finger domain 1 | 1.9296 |
| ***Nuf2*** | "NUF2, NDC80 kinetochore complex component, homolog (S. cerevisiae)" | 1.9291 |
| ***A130009E19Rik*** | RIKEN cDNA A130009E19 gene | 1.9286 |
| ***Ccpg1os*** | "cell cycle progression 1, opposite strand" | 1.9256 |
| ***Smpdl3b*** | "sphingomyelin phosphodiesterase, acid-like 3B" | 1.9236 |
| ***Serpinb1a*** | "serine (or cysteine) peptidase inhibitor, clade B, member 1a" | 1.9191 |
| ***Tceb1*** | "transcription elongation factor B (SIII), polypeptide 1" | 1.9164 |
| ***Ccl9*** | chemokine (C-C motif) ligand 9 | 1.9127 |
| ***Igh-VJ558 /// Igha*** | immunoglobulin heavy chain (J558 family) /// immunoglobulin heavy constant alpha | 1.9102 |
| ***Cdca3*** | cell division cycle associated 3 | 1.9080 |
| ***Gem*** | GTP binding protein (gene overexpressed in skeletal muscle) | 1.9074 |
| ***Lyz2*** | lysozyme 2 | 1.9039 |
| ***Zeb2*** | zinc finger E-box binding homeobox 2 | 1.9032 |
| ***Arsb*** | arylsulfatase B | 1.8987 |
| ***Tmem107*** | transmembrane protein 107 | 1.8975 |
| ***Ttk*** | Ttk protein kinase | 1.8931 |
| ***Ckap2l*** | cytoskeleton associated protein 2-like | 1.8926 |
| ***Ptpn4*** | "protein tyrosine phosphatase, non-receptor type 4" | 1.8882 |
| ***Pmm1*** | phosphomannomutase 1 | 1.8805 |
| ***Immp2l*** | IMP2 inner mitochondrial membrane peptidase-like (S. cerevisiae) | 1.8793 |
| ***Akr1c13*** | "aldo-keto reductase family 1, member C13" | 1.8766 |
| ***Bub1*** | budding uninhibited by benzimidazoles 1 homolog (S. cerevisiae) | 1.8763 |
| ***Gzmm*** | granzyme M (lymphocyte met-ase 1) | 1.8757 |
| ***Prss30*** | "protease, serine 30" | 1.8740 |
| ***Ifitm3*** | interferon induced transmembrane protein 3 | 1.8714 |
| ***Mycbp*** | c-myc binding protein | 1.8707 |
| ***LOC73899*** | uncharacterized LOC73899 | 1.8697 |
| ***Prdx4*** | peroxiredoxin 4 | 1.8663 |
| ***Csf1*** | colony stimulating factor 1 (macrophage) | 1.8636 |
| ***Lcn4*** | lipocalin 4 | 1.8615 |
| ***Map6*** | microtubule-associated protein 6 | 1.8613 |
| ***Cxcr6*** | chemokine (C-X-C motif) receptor 6 | 1.8606 |
| ***Itgb5*** | integrin beta 5 | 1.8601 |
| ***Ighv14-2*** | immunoglobulin heavy variable 14-2 | 1.8573 |
| ***Siva1*** | "SIVA1, apoptosis-inducing factor" | 1.8546 |
| ***Mcoln2*** | mucolipin 2 | 1.8527 |
| ***Ptgr1*** | prostaglandin reductase 1 | 1.8501 |
| ***Gm14005*** | predicted gene 14005 | 1.8500 |
| ***Tm6sf1*** | transmembrane 6 superfamily member 1 | 1.8499 |
| ***Depdc1a*** | DEP domain containing 1a | 1.8497 |
| ***Bco2*** | beta-carotene oxygenase 2 | 1.8472 |
| ***4930562F07Rik*** | RIKEN cDNA 4930562F07 gene | 1.8459 |
| ***2610019E17Rik /// Rab26os*** | "--- /// RAB26, member RAS oncogene family, opposite strand" | 1.8451 |
| ***Lyrm1*** | LYR motif containing 1 | 1.8435 |
| ***Birc5*** | baculoviral IAP repeat-containing 5 | 1.8429 |
| ***Kcnj8*** | "potassium inwardly-rectifying channel, subfamily J, member 8" | 1.8353 |
| ***Hmmr*** | hyaluronan mediated motility receptor (RHAMM) | 1.8332 |
| ***Asns*** | asparagine synthetase | 1.8324 |
| ***Fabp5*** | "fatty acid binding protein 5, epidermal" | 1.8312 |
| ***Lgals3*** | "lectin, galactose binding, soluble 3" | 1.8306 |
| ***5430427O19Rik*** | RIKEN cDNA 5430427O19 gene | 1.8243 |
| ***Ninj2*** | ninjurin 2 | 1.8211 |
| ***Mt1*** | metallothionein 1 | 1.8208 |
| ***Parpbp*** | PARP1 binding protein | 1.8208 |
| ***Car5b*** | "carbonic anhydrase 5b, mitochondrial" | 1.8202 |
| ***Gpx8*** | glutathione peroxidase 8 (putative) | 1.8190 |
| ***Arl6*** | ADP-ribosylation factor-like 6 | 1.8131 |
| ***Il18*** | interleukin 18 | 1.8113 |
| ***Lyz1*** | lysozyme 1 | 1.8094 |
| ***Ccr2*** | chemokine (C-C motif) receptor 2 | 1.8087 |
| ***Fam46a*** | "family with sequence similarity 46, member A" | 1.8084 |
| ***Rfc4*** | replication factor C (activator 1) 4 | 1.8070 |
| ***Acn9*** | ACN9 homolog (S. cerevisiae) | 1.8050 |
| ***Nhsl2*** | NHS-like 2 | 1.8034 |
| ***Gpc1*** | glypican 1 | 1.8006 |
| ***1700012D01Rik /// Tmem194*** | RIKEN cDNA 1700012D01 gene /// transmembrane protein 194 | 1.7933 |
| ***Ncf1*** | neutrophil cytosolic factor 1 | 1.7908 |
| ***Hist1h3a /// Hist1h3b /// Hist1h3c /// Hist1h3d /// Hist1h3e /// Hist1h3f /// Hist1h3g /// Hist1h3h /// Hist1h3i /// Hist2h3b /// Hist2h3c1 /// Hist2h3c2*** | "histone cluster 1, H3a /// histone cluster 1, H3b /// histone cluster 1, H3c /// histone cluster 1, H3d /// histone cluster 1, H3e /// histone cluster 1, H3f /// histone cluster 1, H3g /// histone cluster 1, H3h /// histone cluster 1, H3i /// histone cluster 2, H3b /// histone cluster 2, H3c1 /// histone cluster 2, H3c2" | 1.7898 |
| ***Itga7*** | integrin alpha 7 | 1.7817 |
| ***2610020H08Rik*** | RIKEN cDNA 2610020H08 gene | 1.7803 |
| ***Gm5802*** | predicted gene 5802 | 1.7801 |
| ***Sytl2*** | synaptotagmin-like 2 | 1.7797 |
| ***Prf1*** | perforin 1 (pore forming protein) | 1.7793 |
| ***Bace2*** | beta-site APP-cleaving enzyme 2 | 1.7771 |
| ***Serpinb6a*** | "serine (or cysteine) peptidase inhibitor, clade B, member 6a" | 1.7749 |
| ***Aspm*** | "asp (abnormal spindle)-like, microcephaly associated (Drosophila)" | 1.7716 |
| ***Cks1b*** | CDC28 protein kinase 1b | 1.7699 |
| ***Ckap2*** | cytoskeleton associated protein 2 | 1.7697 |
| ***Tefm*** | "transcription elongation factor, mitochondrial" | 1.7667 |
| ***Pik3r5*** | "phosphoinositide-3-kinase, regulatory subunit 5, p101" | 1.7642 |
| ***Capsl*** | calcyphosine-like | 1.7613 |
| ***Fam151b*** | "family with sequence similarity 151, member B" | 1.7606 |
| ***Rec114*** | REC114 meiotic recombination protein | 1.7600 |
| ***Cdca5*** | cell division cycle associated 5 | 1.7587 |
| ***Spef2*** | sperm flagellar 2 | 1.7573 |
| ***1500009L16Rik*** | RIKEN cDNA 1500009L16 gene | 1.7428 |
| ***St3gal6*** | "ST3 beta-galactoside alpha-2,3-sialyltransferase 6" | 1.7419 |
| ***Timm8a1*** | translocase of inner mitochondrial membrane 8A1 | 1.7373 |
| ***Cerk*** | ceramide kinase | 1.7373 |
| ***1700023H06Rik*** | RIKEN cDNA 1700023H06 gene | 1.7346 |
| ***Fam72a*** | "family with sequence similarity 72, member A" | 1.7324 |
| ***Dscc1*** | defective in sister chromatid cohesion 1 homolog (S. cerevisiae) | 1.7303 |
| ***Enpp4*** | ectonucleotide pyrophosphatase/phosphodiesterase 4 | 1.7289 |
| ***Pbx3*** | pre B cell leukemia homeobox 3 | 1.7272 |
| ***Esm1*** | endothelial cell-specific molecule 1 | 1.7271 |
| ***Lztfl1*** | leucine zipper transcription factor-like 1 | 1.7265 |
| ***2010320M18Rik*** | RIKEN cDNA 2010320M18 gene | 1.7265 |
| ***Ahnak*** | AHNAK nucleoprotein (desmoyokin) | 1.7237 |
| ***Rpf2*** | ribosome production factor 2 homolog (S. cerevisiae) | 1.7208 |
| ***Tk1*** | thymidine kinase 1 | 1.7199 |
| ***Mad2l1*** | MAD2 mitotic arrest deficient-like 1 | 1.7178 |
| ***Dnase1l3*** | deoxyribonuclease 1-like 3 | 1.7170 |
| ***Cttn*** | cortactin | 1.7155 |
| ***Med21*** | mediator complex subunit 21 | 1.7142 |
| ***Rnf13*** | ring finger protein 13 | 1.7117 |
| ***Dkk3*** | dickkopf homolog 3 (Xenopus laevis) | 1.7116 |
| ***Depdc1b*** | DEP domain containing 1B | 1.7094 |
| ***Dbndd2*** | dysbindin (dystrobrevin binding protein 1) domain containing 2 | 1.7083 |
| ***Wdr12*** | WD repeat domain 12 | 1.7069 |
| ***Prdm5*** | PR domain containing 5 | 1.7060 |
| ***Ispd*** | isoprenoid synthase domain containing | 1.7041 |
| ***Itga1*** | integrin alpha 1 | 1.7029 |
| ***Ska1*** | spindle and kinetochore associated complex subunit 1 | 1.7028 |
| ***Fasl*** | "Fas ligand (TNF superfamily, member 6)" | 1.7012 |
| ***Anxa1*** | annexin A1 | 1.7001 |
| ***Grb7*** | growth factor receptor bound protein 7 | 1.6997 |
| ***Extl2*** | exostoses (multiple)-like 2 | 1.6992 |
| ***Wdr37*** | WD repeat domain 37 | 1.6988 |
| ***Sgol2*** | shugoshin-like 2 (S. pombe) | 1.6974 |
| ***Mmp10*** | matrix metallopeptidase 10 | 1.6934 |
| ***C430042M11Rik*** | RIKEN cDNA C430042M11 gene | 1.6927 |
| ***Nhp2*** | NHP2 ribonucleoprotein | 1.6924 |
| ***Rab39b*** | "RAB39B, member RAS oncogene family" | 1.6924 |
| ***Hrsp12*** | heat-responsive protein 12 | 1.6883 |
| ***Dsg2*** | desmoglein 2 | 1.6883 |
| ***Nedd4*** | "neural precursor cell expressed, developmentally down-regulated 4" | 1.6841 |
| ***Gosr1*** | golgi SNAP receptor complex member 1 | 1.6825 |
| ***Diap3*** | diaphanous homolog 3 (Drosophila) | 1.6788 |
| ***Idh3a*** | isocitrate dehydrogenase 3 (NAD+) alpha | 1.6785 |
| ***Tcrg /// Tcrg-C3 /// Tcrg-V4*** | "Mus musculus 2 days neonate thymus thymic cells cDNA, RIKEN full-length enriched library, clone:E430032M10 product:T-cell receptor gamma-1 chain C region homolog [Mus musculus], full insert sequence. /// SubName: Full=Protein Tcrg-C3; /// T cell receptor gamma, variable 4" | 1.6784 |
| ***Slc12a8*** | "solute carrier family 12 (potassium/chloride transporters), member 8" | 1.6775 |
| ***Ptprk*** | "protein tyrosine phosphatase, receptor type, K" | 1.6775 |
| ***Tg*** | thyroglobulin | 1.6769 |
| ***F2r*** | coagulation factor II (thrombin) receptor | 1.6763 |
| ***abParts /// Igkv15-103 /// Igkv15-103*** | "Parts of antibodies, mostly variable regions. /// immunoglobulin kappa chain variable 15-103 /// immunoglobulin kappa chain variable 15-103" | 1.6757 |
| ***Il2ra*** | "interleukin 2 receptor, alpha chain" | 1.6739 |
| ***Orc6*** | "origin recognition complex, subunit 6" | 1.6737 |
| ***Gpr137b-ps*** | "G protein-coupled receptor 137B, pseudogene" | 1.6737 |
| ***Mrpl50*** | mitochondrial ribosomal protein L50 | 1.6723 |
| ***Timm8b*** | translocase of inner mitochondrial membrane 8B | 1.6719 |
| ***Acss2*** | acyl-CoA synthetase short-chain family member 2 | 1.6699 |
| ***S100a1*** | S100 calcium binding protein A1 | 1.6692 |
| ***Dph3*** | diphthamine biosynthesis 3 | 1.6674 |
| ***Celf4*** | "CUGBP, Elav-like family member 4" | 1.6668 |
| ***Csgalnact1*** | chondroitin sulfate N-acetylgalactosaminyltransferase 1 | 1.6664 |
| ***Eps8l1*** | EPS8-like 1 | 1.6654 |
| ***Nacc2*** | "nucleus accumbens associated 2, BEN and BTB (POZ) domain containing" | 1.6615 |
| ***Gmfb*** | "glia maturation factor, beta" | 1.6615 |
| ***1110032A03Rik*** | RIKEN cDNA 1110032A03 gene | 1.6613 |
| ***6330531I01Rik*** | RIKEN cDNA 6330531I01 gene | 1.6607 |
| ***Polr3k*** | polymerase (RNA) III (DNA directed) polypeptide K | 1.6606 |
| ***Kif11*** | kinesin family member 11 | 1.6563 |
| ***Ccnb2*** | cyclin B2 | 1.6559 |
| ***Ttn*** | titin | 1.6549 |
| ***Gpr68*** | G protein-coupled receptor 68 | 1.6523 |
| ***Als2cr12*** | "amyotrophic lateral sclerosis 2 (juvenile) chromosome region, candidate 12 (human)" | 1.6521 |
| ***Tex9*** | testis expressed gene 9 | 1.6510 |
| ***Kif22*** | kinesin family member 22 | 1.6505 |
| ***Dnajc24*** | "DnaJ (Hsp40) homolog, subfamily C, member 24" | 1.6494 |
| ***Echdc2*** | enoyl Coenzyme A hydratase domain containing 2 | 1.6477 |
| ***Ndc80*** | "NDC80 homolog, kinetochore complex component (S. cerevisiae)" | 1.6476 |
| ***4930558N01Rik*** | RIKEN cDNA 4930558N01 gene | 1.6472 |
| ***Fuom*** | fucose mutarotase | 1.6472 |
| ***LOC665506*** | T-cell receptor beta-2 chain C region-like | 1.6469 |
| ***Magohb*** | mago-nashi homolog B (Drosophila) | 1.6435 |
| ***Il1rl1*** | interleukin 1 receptor-like 1 | 1.6425 |
| ***Pcgf1*** | polycomb group ring finger 1 | 1.6421 |
| ***Tnfsf10*** | "tumor necrosis factor (ligand) superfamily, member 10" | 1.6411 |
| ***Tyms /// Tyms-ps*** | "thymidylate synthase /// thymidylate synthase, pseudogene" | 1.6387 |
| ***Deb1*** | differentially expressed in B16F10 1 | 1.6366 |
| ***Crim1*** | cysteine rich transmembrane BMP regulator 1 (chordin like) | 1.6364 |
| ***Ccna2*** | cyclin A2 | 1.6358 |
| ***Gm10451*** | predicted gene 10451 | 1.6347 |
| ***Vmn1r54*** | vomeronasal 1 receptor 54 | 1.6342 |
| ***Cd80*** | CD80 antigen | 1.6341 |
| ***Kif20b*** | kinesin family member 20B | 1.6331 |
| ***Alb*** | albumin | 1.6329 |
| ***Cd24a*** | CD24a antigen | 1.6322 |
| ***Ell2*** | elongation factor RNA polymerase II 2 | 1.6314 |
| ***1110012L19Rik*** | RIKEN cDNA 1110012L19 gene | 1.6298 |
| ***Itga4*** | integrin alpha 4 | 1.6297 |
| ***Coro2a*** | "coronin, actin binding protein 2A" | 1.6286 |
| ***Ccrl2*** | chemokine (C-C motif) receptor-like 2 | 1.6266 |
| ***Rhebl1*** | Ras homolog enriched in brain like 1 | 1.6264 |
| ***Hspa8*** | heat shock protein 8 | 1.6253 |
| ***Prelid2*** | PRELI domain containing 2 | 1.6251 |
| ***Pcyt1a*** | "phosphate cytidylyltransferase 1, choline, alpha isoform" | 1.6237 |
| ***Ngly1*** | N-glycanase 1 | 1.6230 |
| ***Selm*** | selenoprotein M | 1.6202 |
| ***Lactb2*** | "lactamase, beta 2" | 1.6202 |
| ***Rcn1*** | reticulocalbin 1 | 1.6186 |
| ***Wfdc2*** | WAP four-disulfide core domain 2 | 1.6183 |
| ***Sdcbp*** | syndecan binding protein | 1.6181 |
| ***Crem*** | cAMP responsive element modulator | 1.6178 |
| ***Sdcbp2*** | syndecan binding protein (syntenin) 2 | 1.6173 |
| ***Chmp3*** | charged multivesicular body protein 3 | 1.6161 |
| ***Spdl1*** | spindle apparatus coiled-coil protein 1 | 1.6146 |
| ***C1d*** | C1D nuclear receptor co-repressor | 1.6143 |
| ***1700040L02Rik*** | RIKEN cDNA 1700040L02 gene | 1.6132 |
| ***Ifngr1*** | interferon gamma receptor 1 | 1.6130 |
| ***Syce2*** | synaptonemal complex central element protein 2 | 1.6119 |
| ***Pfdn4*** | prefoldin 4 | 1.6113 |
| ***Rrm2*** | ribonucleotide reductase M2 | 1.6111 |
| ***Casp3*** | caspase 3 | 1.6104 |
| ***Vamp7*** | vesicle-associated membrane protein 7 | 1.6103 |
| ***Prr5l*** | proline rich 5 like | 1.6096 |
| ***Apbb1*** | "amyloid beta (A4) precursor protein-binding, family B, member 1" | 1.6092 |
| ***Toporsos*** | "topoisomerase I binding, arginine/serine-rich, opposite strand" | 1.6085 |
| ***Troap*** | trophinin associated protein | 1.6071 |
| ***Usp20*** | ubiquitin specific peptidase 20 | 1.6067 |
| ***Itgae*** | "integrin alpha E, epithelial-associated" | 1.6058 |
| ***2610019N06Rik*** | RIKEN cDNA 2610019N06 gene | 1.6040 |
| ***Serpinb9*** | "serine (or cysteine) peptidase inhibitor, clade B, member 9" | 1.6030 |
| ***Hspa1b*** | heat shock protein 1B | 1.6025 |
| ***Ly6c1 /// Ly6c2*** | "lymphocyte antigen 6 complex, locus C1 /// lymphocyte antigen 6 complex, locus C2" | 1.6004 |
| ***2210015D19Rik*** | RIKEN cDNA 2210015D19 gene | 1.6003 |
| ***F2rl3*** | coagulation factor II (thrombin) receptor-like 3 | 1.6002 |
| ***Cdk5rap1*** | CDK5 regulatory subunit associated protein 1 | 1.5994 |
| ***Ms4a4c*** | "membrane-spanning 4-domains, subfamily A, member 4C" | 1.5993 |
| ***Gadd45g*** | growth arrest and DNA-damage-inducible 45 gamma | 1.5991 |
| ***Pik3cg*** | "phosphoinositide-3-kinase, catalytic, gamma polypeptide" | 1.5981 |
| ***Cenph*** | centromere protein H | 1.5964 |
| ***Nqo2*** | "NAD(P)H dehydrogenase, quinone 2" | 1.5963 |
| ***Hist1h2bc /// Hist1h2be /// Hist1h2bf /// Hist1h2bg /// Hist1h2bj /// Hist1h2bl /// Hist1h2bm /// Hist1h2bn /// Hist1h2bp /// Hist1h2bq /// Hist1h2br*** | "histone cluster 1, H2bc /// histone cluster 1, H2be /// histone cluster 1, H2bf /// histone cluster 1, H2bg /// histone cluster 1, H2bj /// histone cluster 1, H2bl /// histone cluster 1, H2bm /// histone cluster 1, H2bn /// histone cluster 1, H2bp /// histone cluster 1, H2bq /// histone cluster 1 H2br" | 1.5961 |
| ***P2ry14*** | "purinergic receptor P2Y, G-protein coupled, 14" | 1.5953 |
| ***Hist1h2ab /// Hist1h2ac /// Hist1h2ad /// Hist1h2ae /// Hist1h2ag /// Hist1h2ah /// Hist1h2ai /// Hist1h2an /// Hist1h2ao /// Hist1h2ap*** | "histone cluster 1, H2ab /// histone cluster 1, H2ac /// histone cluster 1, H2ad /// histone cluster 1, H2ae /// histone cluster 1, H2ag /// histone cluster 1, H2ah /// histone cluster 1, H2ai /// histone cluster 1, H2an /// histone cluster 1, H2ao /// histone cluster 1, H2ap" | 1.5951 |
| ***Gm13139*** | predicted gene 13139 | 1.5941 |
| ***Xdh*** | xanthine dehydrogenase | 1.5938 |
| ***0610010B08Rik /// Gm14308 /// Gm14326 /// Gm14391 /// Gm14430 /// Gm14434 /// Gm4724 /// LOC102639426*** | RIKEN cDNA 0610010B08 gene /// predicted gene 14308 /// predicted gene 14326 /// predicted gene 14391 /// predicted gene 14430 /// predicted gene 14434 /// predicted gene 4724 /// uncharacterized LOC102639426 | 1.5936 |
| ***Dhrs7*** | dehydrogenase/reductase (SDR family) member 7 | 1.5935 |
| ***Cebpzos*** | "CCAAT/enhancer binding protein (C/EBP), zeta, opposite strand" | 1.5932 |
| ***Gas2l3*** | growth arrest-specific 2 like 3 | 1.5930 |
| ***Mrps22*** | mitochondrial ribosomal protein S22 | 1.5929 |
| ***Dennd5a*** | DENN/MADD domain containing 5A | 1.5912 |
| ***Mrpl35*** | mitochondrial ribosomal protein L35 | 1.5906 |
| ***Sgk3*** | serum/glucocorticoid regulated kinase 3 | 1.5906 |
| ***Snhg1*** | small nucleolar RNA host gene 1 | 1.5906 |
| ***Arl4d*** | ADP-ribosylation factor-like 4D | 1.5903 |
| ***Igf2bp3*** | insulin-like growth factor 2 mRNA binding protein 3 | 1.5901 |
| ***Itih5*** | inter-alpha (globulin) inhibitor H5 | 1.5893 |
| ***Spc24*** | "SPC24, NDC80 kinetochore complex component, homolog (S. cerevisiae)" | 1.5893 |
| ***Fah*** | fumarylacetoacetate hydrolase | 1.5887 |
| ***Gen1*** | "Gen homolog 1, endonuclease (Drosophila)" | 1.5884 |
| ***Efcab7*** | EF-hand calcium binding domain 7 | 1.5884 |
| ***Ndufb2*** | "NADH dehydrogenase (ubiquinone) 1 beta subcomplex, 2" | 1.5882 |
| ***Snhg5*** | small nucleolar RNA host gene 5 | 1.5882 |
| ***Amn1*** | antagonist of mitotic exit network 1 | 1.5880 |
| ***LOC102634034*** | uncharacterized LOC102634034 | 1.5876 |
| ***Cbr4*** | carbonyl reductase 4 | 1.5873 |
| ***Ifitm2*** | interferon induced transmembrane protein 2 | 1.5872 |
| ***Tatdn1*** | TatD DNase domain containing 1 | 1.5867 |
| ***4930453N24Rik*** | RIKEN cDNA 4930453N24 gene | 1.5863 |
| ***Tfpi*** | tissue factor pathway inhibitor | 1.5859 |
| ***Apoo*** | apolipoprotein O | 1.5844 |
| ***Rnaseh2b*** | "ribonuclease H2, subunit B" | 1.5831 |
| ***2010111I01Rik*** | RIKEN cDNA 2010111I01 gene | 1.5820 |
| ***8030451A03Rik*** | RIKEN cDNA 8030451A03 gene | 1.5792 |
| ***Gm561*** | predicted gene 561 | 1.5767 |
| ***Padi2*** | "peptidyl arginine deiminase, type II" | 1.5765 |
| ***Zfp560*** | zinc finger protein 560 | 1.5762 |
| ***Hopx*** | HOP homeobox | 1.5757 |
| ***Fignl1*** | fidgetin-like 1 | 1.5754 |
| ***Nup37*** | nucleoporin 37 | 1.5753 |
| ***Angptl4*** | angiopoietin-like 4 | 1.5752 |
| ***Atg12*** | autophagy related 12 | 1.5752 |
| ***A530054K11Rik*** | RIKEN cDNA A530054K11 gene | 1.5749 |
| ***LOC101055909 /// Sec61b*** | protein transport protein Sec61 subunit beta-like /// Sec61 beta subunit | 1.5744 |
| ***Cdc25c*** | cell division cycle 25C | 1.5743 |
| ***Glmn*** | "glomulin, FKBP associated protein" | 1.5737 |
| ***Ccdc34*** | coiled-coil domain containing 34 | 1.5727 |
| ***Gzmb*** | granzyme B | 1.5722 |
| ***Slc4a1*** | "solute carrier family 4 (anion exchanger), member 1" | 1.5720 |
| ***Tenc1*** | tensin like C1 domain-containing phosphatase | 1.5718 |
| ***Gm9835 /// Gmfg*** | "predicted pseudogene 9835 /// glia maturation factor, gamma" | 1.5713 |
| ***Rpl5 /// Rpl5-ps2*** | "ribosomal protein L5 /// ribisomal protein L5, pseudogene 2" | 1.5712 |
| ***Dtwd1*** | DTW domain containing 1 | 1.5707 |
| ***Tnni3*** | "troponin I, cardiac 3" | 1.5705 |
| ***Cd79b*** | CD79B antigen | 1.5697 |
| ***Dus2*** | dihydrouridine synthase 2 | 1.5692 |
| ***Snhg4*** | small nucleolar RNA host gene 4 | 1.5690 |
| ***1110059E24Rik*** | RIKEN cDNA 1110059E24 gene | 1.5689 |
| ***5830432E09Rik*** | RIKEN cDNA 5830432E09 gene | 1.5685 |
| ***Dancr*** | differentiation antagonizing non-protein coding RNA | 1.5681 |
| ***2210010C04Rik*** | RIKEN cDNA 2210010C04 gene | 1.5677 |
| ***Fastkd2*** | FAST kinase domains 2 | 1.5670 |
| ***Ercc6l*** | excision repair cross-complementing rodent repair deficiency complementation group 6 like | 1.5664 |
| ***Aurka*** | aurora kinase A | 1.5663 |
| ***Znhit3*** | "zinc finger, HIT type 3" | 1.5660 |
| ***Gimap7*** | "GTPase, IMAP family member 7" | 1.5654 |
| ***Tsc22d1*** | "TSC22 domain family, member 1" | 1.5652 |
| ***Eif1a*** | eukaryotic translation initiation factor 1A | 1.5649 |
| ***Pmaip1*** | phorbol-12-myristate-13-acetate-induced protein 1 | 1.5645 |
| ***Rad54b*** | RAD54 homolog B (S. cerevisiae) | 1.5644 |
| ***Melk*** | maternal embryonic leucine zipper kinase | 1.5641 |
| ***9030617O03Rik*** | RIKEN cDNA 9030617O03 gene | 1.5640 |
| ***H2-Ob*** | "histocompatibility 2, O region beta locus" | 1.5639 |
| ***5830468F06Rik*** | RIKEN cDNA 5830468F06 gene | 1.5638 |
| ***Gab3*** | growth factor receptor bound protein 2-associated protein 3 | 1.5632 |
| ***Cdadc1*** | cytidine and dCMP deaminase domain containing 1 | 1.5630 |
| ***Rpp40*** | ribonuclease P 40 subunit | 1.5619 |
| ***Tmbim4*** | transmembrane BAX inhibitor motif containing 4 | 1.5611 |
| ***Themis2*** | thymocyte selection associated family member 2 | 1.5606 |
| ***Slc43a1*** | "solute carrier family 43, member 1" | 1.5604 |
| ***Ccdc58*** | coiled-coil domain containing 58 | 1.5595 |
| ***Ptrh2*** | peptidyl-tRNA hydrolase 2 | 1.5593 |
| ***Tpx2*** | "TPX2, microtubule-associated protein homolog (Xenopus laevis)" | 1.5582 |
| ***Acoxl*** | acyl-Coenzyme A oxidase-like | 1.5563 |
| ***Ptrhd1*** | peptidyl-tRNA hydrolase domain containing 1 | 1.5561 |
| ***Gm17748 /// Ppih*** | "predicted gene, 17748 /// peptidyl prolyl isomerase H" | 1.5559 |
| ***Ska2*** | spindle and kinetochore associated complex subunit 2 | 1.5556 |
| ***Rpp30*** | ribonuclease P/MRP 30 subunit | 1.5545 |
| ***Arhgap15*** | Rho GTPase activating protein 15 | 1.5545 |
| ***Abhd14a*** | abhydrolase domain containing 14A | 1.5538 |
| ***Atp13a3*** | ATPase type 13A3 | 1.5528 |
| ***Fam213b*** | "family with sequence similarity 213, member B" | 1.5526 |
| ***Pole3*** | "polymerase (DNA directed), epsilon 3 (p17 subunit)" | 1.5526 |
| ***Dpcd*** | deleted in primary ciliary dyskinesia | 1.5521 |
| ***Zfp958*** | zinc finger protein 958 | 1.5518 |
| ***Hrh2*** | histamine receptor H2 | 1.5511 |
| ***Rom1*** | rod outer segment membrane protein 1 | 1.5508 |
| ***Pbk*** | PDZ binding kinase | 1.5506 |
| ***9430038I01Rik*** | RIKEN cDNA 9430038I01 gene | 1.5495 |
| ***Uxt*** | ubiquitously expressed transcript | 1.5485 |
| ***H2afz*** | "H2A histone family, member Z" | 1.5484 |
| ***Osgepl1*** | O-sialoglycoprotein endopeptidase-like 1 | 1.5478 |
| ***Chek1*** | checkpoint kinase 1 | 1.5477 |
| ***Kirrel3*** | kin of IRRE like 3 (Drosophila) | 1.5467 |
| ***Cobll1*** | Cobl-like 1 | 1.5467 |
| ***Tardbp*** | TAR DNA binding protein | 1.5464 |
| ***Lst1*** | leukocyte specific transcript 1 | 1.5459 |
| ***Crip2*** | cysteine rich protein 2 | 1.5453 |
| ***Traip*** | TRAF-interacting protein | 1.5452 |
| ***4930562C15Rik*** | RIKEN cDNA 4930562C15 gene | 1.5436 |
| ***Chchd5*** | coiled-coil-helix-coiled-coil-helix domain containing 5 | 1.5430 |
| ***Gmnn*** | geminin | 1.5429 |
| ***Tyrobp*** | TYRO protein tyrosine kinase binding protein | 1.5420 |
| ***Erich3*** | glutamate rich 3 | 1.5414 |
| ***Gtf2f2*** | "general transcription factor IIF, polypeptide 2" | 1.5404 |
| ***Tacc3*** | "transforming, acidic coiled-coil containing protein 3" | 1.5393 |
| ***Smim4*** | small itegral membrane protein 4 | 1.5390 |
| ***Gpr137b /// Gpr137b-ps*** | "G protein-coupled receptor 137B /// G protein-coupled receptor 137B, pseudogene" | 1.5381 |
| ***Uba3*** | ubiquitin-like modifier activating enzyme 3 | 1.5377 |
| ***Kank3*** | KN motif and ankyrin repeat domains 3 | 1.5377 |
| ***Anln*** | "anillin, actin binding protein" | 1.5375 |
| ***Enpp1*** | ectonucleotide pyrophosphatase/phosphodiesterase 1 | 1.5366 |
| ***9030619P08Rik*** | lymphocyte antigen 6 complex pseudogene | 1.5355 |
| ***Eomes*** | eomesodermin homolog (Xenopus laevis) | 1.5353 |
| ***Env*** | uncharacterized LOC641050 | 1.5350 |
| ***Camk2n1*** | calcium/calmodulin-dependent protein kinase II inhibitor 1 | 1.5347 |
| ***Elovl7*** | "ELOVL family member 7, elongation of long chain fatty acids (yeast)" | 1.5333 |
| ***1700020I14Rik*** | RIKEN cDNA 1700020I14 gene | 1.5331 |
| ***Mylpf*** | "myosin light chain, phosphorylatable, fast skeletal muscle" | 1.5326 |
| ***Mtus2*** | microtubule associated tumor suppressor candidate 2 | 1.5324 |
| ***Clip2*** | CAP-GLY domain containing linker protein 2 | 1.5320 |
| ***Arhgap19*** | Rho GTPase activating protein 19 | 1.5319 |
| ***Anapc15*** | anaphase prompoting complex C subunit 15 | 1.5314 |
| ***Rad51b*** | RAD51 homolog B | 1.5309 |
| ***Ncapg2*** | "non-SMC condensin II complex, subunit G2" | 1.5308 |
| ***Mrpl48*** | mitochondrial ribosomal protein L48 | 1.5300 |
| ***5031425E22Rik*** | RIKEN cDNA 5031425E22 gene | 1.5300 |
| ***LOC553093*** | uncharacterized LOC553093 | 1.5299 |
| ***5530601H04Rik*** | RIKEN cDNA 5530601H04 gene | 1.5298 |
| ***Ahsg*** | alpha-2-HS-glycoprotein | 1.5294 |
| ***Fcf1*** | FCF1 small subunit (SSU) processome component homolog (S. cerevisiae) | 1.5289 |
| ***Ccl6*** | chemokine (C-C motif) ligand 6 | 1.5288 |
| ***Zbtb32*** | zinc finger and BTB domain containing 32 | 1.5285 |
| ***Ryr3*** | ryanodine receptor 3 | 1.5283 |
| ***Gm6851 /// Gm7301 /// Pin4*** | "predicted pseudogene 6851 /// predicted gene 7301 /// protein (peptidyl-prolyl cis/trans isomerase) NIMA-interacting, 4 (parvulin)" | 1.5282 |
| ***Galr3 /// Gcat*** | galanin receptor 3 /// glycine C-acetyltransferase (2-amino-3-ketobutyrate-coenzyme A ligase) | 1.5277 |
| ***Cops5*** | "COP9 (constitutive photomorphogenic) homolog, subunit 5 (Arabidopsis thaliana)" | 1.5256 |
| ***Eny2*** | enhancer of yellow 2 homolog (Drosophila) | 1.5240 |
| ***Zfp949*** | zinc finger protein 949 | 1.5239 |
| ***Cd8b1*** | "CD8 antigen, beta chain 1" | 1.5229 |
| ***10-Sep*** | septin 10 | 1.5228 |
| ***Tmem237*** | transmembrane protein 237 | 1.5222 |
| ***Pigf*** | "phosphatidylinositol glycan anchor biosynthesis, class F" | 1.5220 |
| ***Prc1*** | protein regulator of cytokinesis 1 | 1.5216 |
| ***Ccdc173*** | coiled-coil domain containing 173 | 1.5214 |
| ***Ift74*** | intraflagellar transport 74 | 1.5213 |
| ***Thyn1*** | thymocyte nuclear protein 1 | 1.5208 |
| ***Ercc6l2*** | "excision repair cross-complementing rodent repair deficiency, complementation group 6 like 2" | 1.5197 |
| ***Psmc3ip*** | "proteasome (prosome, macropain) 26S subunit, ATPase 3, interacting protein" | 1.5195 |
| ***Adam6a /// Adam6b*** | a disintegrin and metallopeptidase domain 6A /// a disintegrin and metallopeptidase domain 6B | 1.5186 |
| ***Slamf7*** | SLAM family member 7 | 1.5186 |
| ***Nudt7*** | nudix (nucleoside diphosphate linked moiety X)-type motif 7 | 1.5181 |
| ***Il10ra*** | "interleukin 10 receptor, alpha" | 1.5179 |
| ***Pdp1*** | pyruvate dehyrogenase phosphatase catalytic subunit 1 | 1.5178 |
| ***Zswim7*** | zinc finger SWIM-type containing 7 | 1.5162 |
| ***Chst11*** | carbohydrate sulfotransferase 11 | 1.5155 |
| ***Insl6*** | insulin-like 6 | 1.5144 |
| ***Nek2*** | NIMA (never in mitosis gene a)-related expressed kinase 2 | 1.5140 |
| ***Acsbg1*** | acyl-CoA synthetase bubblegum family member 1 | 1.5130 |
| ***Mcm7*** | minichromosome maintenance deficient 7 (S. cerevisiae) | 1.5126 |
| ***Mbd1*** | methyl-CpG binding domain protein 1 | 1.5126 |
| ***AA408251*** | expressed sequence AA408251 | 1.5122 |
| ***H2-Ob /// LOC102633400*** | "histocompatibility 2, O region beta locus /// uncharacterized LOC102633400" | 1.5120 |
| ***Tacstd2*** | tumor-associated calcium signal transducer 2 | 1.5116 |
| ***1700084F23Rik*** | RIKEN cDNA 1700084F23 gene | 1.5114 |
| ***Mtch2*** | mitochondrial carrier homolog 2 (C. elegans) | 1.5109 |
| ***Tec*** | tec protein tyrosine kinase | 1.5108 |
| ***2810001G20Rik*** | RIKEN cDNA 2810001G20 gene | 1.5106 |
| ***Gm14446*** | predicted gene 14446 | 1.5101 |
| ***Ugcg*** | UDP-glucose ceramide glucosyltransferase | 1.5098 |
| ***Slirp*** | SRA stem-loop interacting RNA binding protein | 1.5096 |
| ***B230307C23Rik*** | RIKEN cDNA B230307C23 gene | 1.5093 |
| ***Itgb1bp1*** | integrin beta 1 binding protein 1 | 1.5092 |
| ***Hrh4*** | histamine receptor H4 | 1.5091 |
| ***Mkks*** | McKusick-Kaufman syndrome | 1.5088 |
| ***Ccng1*** | cyclin G1 | 1.5087 |
| ***Cks2*** | CDC28 protein kinase regulatory subunit 2 | 1.5085 |
| ***S100a13*** | S100 calcium binding protein A13 | 1.5084 |
| ***Smc2*** | structural maintenance of chromosomes 2 | 1.5079 |
| ***2310009A05Rik*** | RIKEN cDNA 2310009A05 gene | 1.5079 |
| ***Prdm1*** | "PR domain containing 1, with ZNF domain" | 1.5074 |
| ***Ndufaf2*** | "NADH dehydrogenase (ubiquinone) 1 alpha subcomplex, assembly factor 2" | 1.5073 |
| ***1700007L15Rik*** | RIKEN cDNA 1700007L15 gene | 1.5063 |
| ***Btf3l4*** | basic transcription factor 3-like 4 | 1.5060 |
| ***Cnrip1*** | cannabinoid receptor interacting protein 1 | 1.5059 |
| ***Scfd1*** | Sec1 family domain containing 1 | 1.5053 |
| ***4930523C07Rik*** | RIKEN cDNA 4930523C07 gene | 1.5042 |
| ***Tsacc*** | TSSK6 activating co-chaperone | 1.5037 |
| ***Cdc45*** | cell division cycle 45 | 1.5030 |
| ***1700097N02Rik*** | RIKEN cDNA 1700097N02 gene | 1.5029 |
| ***Cxcl10*** | chemokine (C-X-C motif) ligand 10 | 1.5025 |
| ***Zgrf1*** | "zinc finger, GRF-type containing 1" | 1.5025 |
| ***Coa5*** | cytochrome C oxidase assembly factor 5 | 1.5019 |
| ***Rpain*** | RPA interacting protein | 1.5019 |
| ***Mrpl1*** | mitochondrial ribosomal protein L1 | 1.5019 |
| ***Prss2*** | "protease, serine 2" | 1.5018 |
| ***Zfp930*** | zinc finger protein 930 | 1.5014 |
| ***Gm9895*** | predicted gene 9895 | 1.5009 |
| ***Nme7*** | NME/NM23 family member 7 | 1.5008 |
| ***Gins1*** | GINS complex subunit 1 (Psf1 homolog) | 1.5007 |
| ***Il21r*** | interleukin 21 receptor | -1.2968 |
| ***Il6st*** | interleukin 6 signal transducer | -1.4380 |
| ***Pdcd1*** | programmed cell death 1 | -1.4830 |
| ***Tcf7*** | "transcription factor 7, T cell specific" | -1.4861 |
| ***Acsl3*** | acyl-CoA synthetase long-chain family member 3 | -1.4957 |
| ***Senp6*** | SUMO/sentrin specific peptidase 6 | -1.5006 |
| ***6030439D06Rik*** | RIKEN cDNA 6030439D06 gene | -1.5015 |
| ***Setd1b*** | SET domain containing 1B | -1.5024 |
| ***Grn*** | granulin | -1.5029 |
| ***4930573C08Rik*** | RIKEN cDNA 4930573C08 gene | -1.5033 |
| ***Fam71b*** | "family with sequence similarity 71, member B" | -1.5038 |
| ***Map1lc3b*** | microtubule-associated protein 1 light chain 3 beta | -1.5040 |
| ***Egr2*** | early growth response 2 | -1.5044 |
| ***Jmjd1c*** | jumonji domain containing 1C | -1.5053 |
| ***Gda*** | guanine deaminase | -1.5078 |
| ***Slc5a3*** | "solute carrier family 5 (inositol transporters), member 3" | -1.5083 |
| ***Ddx6*** | DEAD (Asp-Glu-Ala-Asp) box polypeptide 6 | -1.5090 |
| ***Sufu*** | suppressor of fused homolog (Drosophila) | -1.5108 |
| ***Gm19313*** | "predicted gene, 19313" | -1.5110 |
| ***Ptprc*** | "protein tyrosine phosphatase, receptor type, C" | -1.5119 |
| ***A130071D04Rik*** | RIKEN cDNA A130071D04 gene | -1.5124 |
| ***Btrc*** | beta-transducin repeat containing protein | -1.5140 |
| ***Plxnd1*** | plexin D1 | -1.5156 |
| ***Sesn3*** | sestrin 3 | -1.5163 |
| ***Ddx58*** | DEAD (Asp-Glu-Ala-Asp) box polypeptide 58 | -1.5177 |
| ***Gm3776 /// Gsta1 /// Gsta2*** | "predicted gene 3776 /// glutathione S-transferase, alpha 1 (Ya) /// glutathione S-transferase, alpha 2 (Yc2)" | -1.5191 |
| ***Rsad2*** | radical S-adenosyl methionine domain containing 2 | -1.5198 |
| ***6430598H11Rik*** | RIKEN cDNA 6430598H11 gene | -1.5198 |
| ***Jade2*** | jade family PHD finger 2 | -1.5200 |
| ***Kdm5a*** | lysine (K)-specific demethylase 5A | -1.5207 |
| ***D730035F11Rik*** | RIKEN cDNA D730035F11 gene | -1.5207 |
| ***AK164218*** | "Mus musculus 16 days embryo head cDNA, RIKEN full-length enriched library, clone:C130022D12 product:unclassifiable, full insert sequence." | -1.5221 |
| ***Ext1*** | exostoses (multiple) 1 | -1.5223 |
| ***Slc7a11*** | "solute carrier family 7 (cationic amino acid transporter, y+ system), member 11" | -1.5235 |
| ***Gm10340 /// Gm10409 /// Gm16525 /// Gm2897 /// Gm3002 /// Gm3005 /// Gm3095 /// Gm3115 /// Gm3164 /// Gm3173 /// Gm3239 /// Gm3252 /// Gm3264 /// Gm3317 /// Gm3373 /// Gm3383 /// Gm3488 /// Gm3500 /// Gm3558 /// Gm3636 /// Gm3642 /// Gm3667 /// Gm3696 /// Gm3739 /// Gm5796 /// LOC100861615 /// LOC102638110*** | "predicted gene 10340 /// predicted pseudogene 8348 /// predicted gene, 16525 /// predicted gene 2897 /// alpha-takusan pseudogene /// predicted gene 3005 /// predicted gene 3095 /// predicted gene 3115 /// predicted gene 3164 /// predicted gene 3173 /// predicted gene 3239 /// predicted gene 3252 /// predicted gene 3264 /// predicted gene 3317 /// predicted gene 3373 /// predicted gene 3383 /// predicted gene, 3488 /// predicted gene 3500 /// predicted gene 3558 /// predicted gene 3636 /// predicted gene 3642 /// predicted gene 3667 /// predicted gene 3696 /// predicted gene 3739 /// predicted gene 5796 /// alpha takusan-like /// uncharacterized LOC102638110" | -1.5251 |
| ***Tmem178b*** | transmembrane protein 178B | -1.5270 |
| ***Pptc7*** | PTC7 protein phosphatase homolog (S. cerevisiae) | -1.5291 |
| ***2010007H06Rik /// LOC102635638*** | RIKEN cDNA 2010007H06 gene /// uncharacterized LOC102635638 | -1.5302 |
| ***Stxbp1*** | syntaxin binding protein 1 | -1.5305 |
| ***Pip4k2b*** | "phosphatidylinositol-5-phosphate 4-kinase, type II, beta" | -1.5309 |
| ***Hoxb5os*** | "homeobox B5 and homeobox B6, opposite strand" | -1.5330 |
| ***Ebpl*** | emopamil binding protein-like | -1.5342 |
| ***Ifi44*** | interferon-induced protein 44 | -1.5344 |
| ***Il6ra*** | "interleukin 6 receptor, alpha" | -1.5356 |
| ***Git2*** | G protein-coupled receptor kinase-interactor 2 | -1.5361 |
| ***Klf12*** | Kruppel-like factor 12 | -1.5366 |
| ***Serpina9*** | "serine (or cysteine) peptidase inhibitor, clade A (alpha-1 antiproteinase, antitrypsin), member 9" | -1.5378 |
| ***Osbpl9*** | oxysterol binding protein-like 9 | -1.5382 |
| ***Dnajb2*** | "DnaJ (Hsp40) homolog, subfamily B, member 2" | -1.5389 |
| ***Zbtb20*** | zinc finger and BTB domain containing 20 | -1.5396 |
| ***Plekhg2*** | "pleckstrin homology domain containing, family G (with RhoGef domain) member 2" | -1.5406 |
| ***Huwe1*** | "HECT, UBA and WWE domain containing 1" | -1.5406 |
| ***Dock4*** | dedicator of cytokinesis 4 | -1.5425 |
| ***Camk4*** | calcium/calmodulin-dependent protein kinase IV | -1.5449 |
| ***Rnf128*** | ring finger protein 128 | -1.5449 |
| ***4930529C04Rik /// Gm15583 /// Zbed4*** | "zinc finger, BED domain containing 4 pseudogene /// predicted gene 15583 /// zinc finger, BED domain containing 4" | -1.5470 |
| ***Tnik*** | TRAF2 and NCK interacting kinase | -1.5482 |
| ***Arrb1*** | "arrestin, beta 1" | -1.5485 |
| ***Synj2*** | synaptojanin 2 | -1.5492 |
| ***5830453J16Rik*** | RIKEN cDNA 5830453J16 gene | -1.5501 |
| ***Iffo2*** | intermediate filament family orphan 2 | -1.5506 |
| ***Ikzf2*** | IKAROS family zinc finger 2 | -1.5511 |
| ***Grhl1*** | grainyhead-like 1 (Drosophila) | -1.5523 |
| ***1110018F16Rik /// Dnajb14*** | "RIKEN cDNA 1110018F16 gene /// DnaJ (Hsp40) homolog, subfamily B, member 14" | -1.5528 |
| ***A130001G05Rik*** | RIKEN cDNA A130001G05 gene | -1.5540 |
| ***Snn*** | stannin | -1.5545 |
| ***Atf7ip*** | activating transcription factor 7 interacting protein | -1.5550 |
| ***Snx5*** | sorting nexin 5 | -1.5552 |
| ***Actr1a*** | "ARP1 actin-related protein 1A, centractin alpha" | -1.5567 |
| ***Cdk5r1*** | "cyclin-dependent kinase 5, regulatory subunit 1 (p35)" | -1.5584 |
| ***Mafg*** | "v-maf musculoaponeurotic fibrosarcoma oncogene family, protein G (avian)" | -1.5591 |
| ***Ccdc7*** | coiled-coil domain containing 7 | -1.5591 |
| ***Trim8*** | tripartite motif-containing 8 | -1.5605 |
| ***Malt1*** | mucosa associated lymphoid tissue lymphoma translocation gene 1 | -1.5627 |
| ***6330575P09Rik*** | RIKEN cDNA 6330575P09 gene | -1.5632 |
| ***Btg1*** | "B cell translocation gene 1, anti-proliferative" | -1.5647 |
| ***Rbm39*** | RNA binding motif protein 39 | -1.5652 |
| ***Csprs /// Gm15433 /// Gm2666 /// Gm7609 /// LOC100041903 /// LOC100503923*** | component of Sp100-rs /// predicted pseudogene 15433 /// predicted gene 2666 /// predicted pseudogene 7609 /// uncharacterized LOC100041903 /// uncharacterized LOC100503923 | -1.5674 |
| ***Klf4*** | Kruppel-like factor 4 (gut) | -1.5681 |
| ***Cers6*** | ceramide synthase 6 | -1.5706 |
| ***Bcl11b*** | B cell leukemia/lymphoma 11B | -1.5733 |
| ***Klk1b27*** | kallikrein 1-related peptidase b27 | -1.5733 |
| ***AU019559*** | expressed sequence AU019559 | -1.5738 |
| ***Egr1*** | early growth response 1 | -1.5751 |
| ***Kmt2a*** | lysine (K)-specific methyltransferase 2A | -1.5758 |
| ***Tnfsf14*** | "tumor necrosis factor (ligand) superfamily, member 14" | -1.5773 |
| ***Sik1*** | salt inducible kinase 1 | -1.5783 |
| ***Usp6nl*** | USP6 N-terminal like | -1.5795 |
| ***Sh3rf1*** | SH3 domain containing ring finger 1 | -1.5803 |
| ***Hif1a*** | "hypoxia inducible factor 1, alpha subunit" | -1.5815 |
| ***Usp4*** | ubiquitin specific peptidase 4 (proto-oncogene) | -1.5815 |
| ***Mir1931*** | microRNA 1931 | -1.5853 |
| ***Tox*** | thymocyte selection-associated high mobility group box | -1.5858 |
| ***Cebpa*** | "CCAAT/enhancer binding protein (C/EBP), alpha" | -1.5883 |
| ***Dip2b*** | DIP2 disco-interacting protein 2 homolog B (Drosophila) | -1.5883 |
| ***Zfp467*** | zinc finger protein 467 | -1.5886 |
| ***1810006J02Rik*** | RIKEN cDNA 1810006J02 gene | -1.5888 |
| ***Jdp2*** | Jun dimerization protein 2 | -1.5898 |
| ***Fos*** | FBJ osteosarcoma oncogene | -1.5921 |
| ***Kdm6b*** | KDM1 lysine (K)-specific demethylase 6B | -1.5934 |
| ***AK088937*** | "Mus musculus adult male thymus cDNA, RIKEN full-length enriched library, clone:5830436I19 product:unclassifiable, full insert sequence." | -1.5939 |
| ***Ddx3y*** | "DEAD (Asp-Glu-Ala-Asp) box polypeptide 3, Y-linked" | -1.5941 |
| ***Stk39*** | serine/threonine kinase 39 | -1.5977 |
| ***Fgf13*** | fibroblast growth factor 13 | -1.5977 |
| ***Trp53bp1*** | transformation related protein 53 binding protein 1 | -1.6000 |
| ***Akna*** | AT-hook transcription factor | -1.6064 |
| ***Pacsin1*** | protein kinase C and casein kinase substrate in neurons 1 | -1.6067 |
| ***Dtx1*** | deltex 1 homolog (Drosophila) | -1.6090 |
| ***Wnt10a*** | "wingless-type MMTV integration site family, member 10A" | -1.6098 |
| ***Nrip1*** | nuclear receptor interacting protein 1 | -1.6113 |
| ***Malat1*** | metastasis associated lung adenocarcinoma transcript 1 (non-coding RNA) | -1.6134 |
| ***Foxo1*** | forkhead box O1 | -1.6142 |
| ***Vopp1*** | "vesicular, overexpressed in cancer, prosurvival protein 1" | -1.6147 |
| ***Ramp3*** | receptor (calcitonin) activity modifying protein 3 | -1.6155 |
| ***Ass1 /// Gm5424*** | argininosuccinate synthetase 1 /// argininosuccinate synthase pseudogene | -1.6176 |
| ***Gm12789*** | predicted gene 12789 | -1.6179 |
| ***C78339*** | expressed sequence C78339 | -1.6234 |
| ***Rap2b*** | "RAP2B, member of RAS oncogene family" | -1.6258 |
| ***Folr4*** | folate receptor 4 (delta) | -1.6273 |
| ***Rnase4*** | "ribonuclease, RNase A family 4" | -1.6303 |
| ***Laptm5*** | lysosomal-associated protein transmembrane 5 | -1.6308 |
| ***Tspan13*** | tetraspanin 13 | -1.6332 |
| ***Tubb2a*** | "tubulin, beta 2A class IIA" | -1.6367 |
| ***Zbtb18*** | zinc finger and BTB domain containing 18 | -1.6396 |
| ***Atg13*** | autophagy related 13 | -1.6404 |
| ***Angptl2*** | angiopoietin-like 2 | -1.6434 |
| ***Cxcr5*** | chemokine (C-X-C motif) receptor 5 | -1.6442 |
| ***Araf*** | v-raf murine sarcoma 3611 viral oncogene homolog | -1.6485 |
| ***Dmwd*** | dystrophia myotonica-containing WD repeat motif | -1.6551 |
| ***D1Ertd646e*** | "DNA segment, Chr 1, ERATO Doi 646, expressed" | -1.6565 |
| ***Ascl2*** | achaete-scute complex homolog 2 (Drosophila) | -1.6578 |
| ***Zfp36l1*** | "zinc finger protein 36, C3H type-like 1" | -1.6592 |
| ***Pabpc1*** | "poly(A) binding protein, cytoplasmic 1" | -1.6603 |
| ***Dnmt3a*** | DNA methyltransferase 3A | -1.6664 |
| ***Setd4*** | SET domain containing 4 | -1.6700 |
| ***4933402N22Rik /// Gm5861 /// Gm6460 /// Speer1-ps1*** | "RIKEN cDNA 4933402N22 gene /// predicted gene 5861 /// predicted gene 6460 /// spermatogenesis associated glutamate (E)-rich protein 1, pseudogene 1" | -1.6700 |
| ***5430421N21Rik*** | RIKEN cDNA 5430421N21 gene | -1.6734 |
| ***Acvr1b*** | "activin A receptor, type 1B" | -1.6736 |
| ***Matk*** | megakaryocyte-associated tyrosine kinase | -1.6776 |
| ***Ephx1*** | "epoxide hydrolase 1, microsomal" | -1.6790 |
| ***D15Wsu126e*** | "DNA segment, Chr 15, Wayne State University 126, expressed" | -1.6863 |
| ***Irf2bpl*** | interferon regulatory factor 2 binding protein-like | -1.6886 |
| ***Dpy19l3*** | dpy-19-like 3 (C. elegans) | -1.6903 |
| ***9430082L08Rik*** | RIKEN cDNA 9430082L08 gene | -1.6958 |
| ***Abtb2*** | ankyrin repeat and BTB (POZ) domain containing 2 | -1.6972 |
| ***Ak4*** | adenylate kinase 4 | -1.7033 |
| ***Ccar1*** | cell division cycle and apoptosis regulator 1 | -1.7039 |
| ***Gcg*** | glucagon | -1.7164 |
| ***Smox*** | spermine oxidase | -1.7188 |
| ***Fam101b*** | "family with sequence similarity 101, member B" | -1.7200 |
| ***B4galnt4*** | "beta-1,4-N-acetyl-galactosaminyl transferase 4" | -1.7206 |
| ***Nfatc1*** | "nuclear factor of activated T cells, cytoplasmic, calcineurin dependent 1" | -1.7334 |
| ***Trav12n-2*** | T cell receptor alpha variable 12N-2 | -1.7334 |
| ***Rhoh*** | "ras homolog gene family, member H" | -1.7501 |
| ***Padi4*** | "peptidyl arginine deiminase, type IV" | -1.7768 |
| ***Plekho1*** | "pleckstrin homology domain containing, family O member 1" | -1.7775 |
| ***Abcb9*** | "ATP-binding cassette, sub-family B (MDR/TAP), member 9" | -1.7794 |
| ***Otud4*** | OTU domain containing 4 | -1.7813 |
| ***Hivep3*** | human immunodeficiency virus type I enhancer binding protein 3 | -1.7813 |
| ***Ntn1*** | netrin 1 | -1.7822 |
| ***Sfmbt2*** | Scm-like with four mbt domains 2 | -1.7851 |
| ***Trp53inp2*** | transformation related protein 53 inducible nuclear protein 2 | -1.7873 |
| ***Fmnl3*** | formin-like 3 | -1.7902 |
| ***Phactr2*** | phosphatase and actin regulator 2 | -1.7937 |
| ***LOC102641248 /// Maf*** | uncharacterized LOC102641248 /// avian musculoaponeurotic fibrosarcoma (v-maf) AS42 oncogene homolog | -1.8028 |
| ***St6gal1*** | "beta galactoside alpha 2,6 sialyltransferase 1" | -1.8041 |
| ***Tox2*** | TOX high mobility group box family member 2 | -1.8060 |
| ***Stk17b*** | serine/threonine kinase 17b (apoptosis-inducing) | -1.8205 |
| ***Sema7a*** | "sema domain, immunoglobulin domain (Ig), and GPI membrane anchor, (semaphorin) 7A" | -1.8430 |
| ***Tbc1d2b*** | "TBC1 domain family, member 2B" | -1.8567 |
| ***Fam43a*** | "family with sequence similarity 43, member A" | -1.8570 |
| ***Runx1*** | runt related transcription factor 1 | -1.8699 |
| ***Tesc*** | tescalcin | -1.8929 |
| ***Il21*** | interleukin 21 | -1.8968 |
| ***Cd22*** | CD22 antigen | -1.9305 |
| ***Klhl4*** | kelch-like 4 | -1.9414 |
| ***Maf*** | avian musculoaponeurotic fibrosarcoma (v-maf) AS42 oncogene homolog | -1.9482 |
| ***Asb2*** | ankyrin repeat and SOCS box-containing 2 | -1.9865 |
| ***Bcl6*** | B cell leukemia/lymphoma 6 | -1.9916 |
| ***BB163080*** | expressed sequence BB163080 | -2.0024 |
| ***A430107P09Rik*** | RIKEN cDNA A430107P09 gene | -2.0080 |
| ***Lpp*** | LIM domain containing preferred translocation partner in lipoma | -2.0145 |
| ***Penk*** | preproenkephalin | -2.0210 |
| ***Tnfsf11*** | "tumor necrosis factor (ligand) superfamily, member 11" | -2.0272 |
| ***Rsrp1*** | arginine/serine rich protein 1 | -2.0276 |
| ***Timp2*** | tissue inhibitor of metalloproteinase 2 | -2.0325 |
| ***Eif4h*** | eukaryotic translation initiation factor 4H | -2.0387 |
| ***Afap1*** | actin filament associated protein 1 | -2.0717 |
| ***2900064B18Rik*** | RIKEN cDNA 2900064B18 gene | -2.0803 |
| ***Ncor1*** | nuclear receptor co-repressor 1 | -2.1542 |
| ***P2rx7*** | "purinergic receptor P2X, ligand-gated ion channel, 7" | -2.1839 |
| ***Ccr6*** | chemokine (C-C motif) receptor 6 | -2.2257 |
| ***4833423F13Rik*** | RIKEN cDNA 4833423F13 gene | -2.2645 |
| ***Ramp1*** | receptor (calcitonin) activity modifying protein 1 | -2.2936 |
| ***Egln3*** | egl-9 family hypoxia-inducible factor 3 | -2.3552 |
| ***Coro2b*** | "coronin, actin binding protein, 2B" | -2.3629 |
| ***Rnf144a*** | ring finger protein 144A | -2.3987 |
| ***Zfp142*** | zinc finger protein 142 | -2.4010 |
| ***Tgfbr3*** | "transforming growth factor, beta receptor III" | -2.5284 |
| ***Ddit4*** | DNA-damage-inducible transcript 4 | -2.6518 |
| ***Nav2*** | neuron navigator 2 | -2.6724 |
| ***Spsb1*** | splA/ryanodine receptor domain and SOCS box containing 1 | -2.6925 |
| ***Ntrk3*** | "neurotrophic tyrosine kinase, receptor, type 3" | -2.9481 |
| ***Sox4*** | SRY (sex determining region Y)-box 4 | -2.9595 |
| ***Xaf1*** | XIAP associated factor 1 | -3.8124 |
| ***H2-Q2*** | "histocompatibility 2, Q region locus 2" | -4.2589 |

Data represents the average of 2 independent samples of *Rictor*^-/-^ and WT T_FH_ cells analyzed by microarray. Genes with 1.5-fold change cutoff (upregulated and downregulated) in *Rictor*^-/-^ T_FH_ cells are listed in the table. Positive fold changes indicate upregulated genes and negative fold changes indicate downregulated genes in *Rictor*^-/-^ T_FH_ cells. Genes highlighted in yellow are genes of interest.
